# Emergence of Rechargeable Aqueous Manganese Batteries

**DOI:** 10.1007/s40820-026-02174-z

**Published:** 2026-04-08

**Authors:** Zikang Xu, Lifen Long, Ying Yang, Zeyu Cao, Hang Ren, Zhixuan Wei, Laifa Shen, Heng Jiang, Huaiyu Shao, Shengyang Dong

**Affiliations:** 1 Jiangsu Key Laboratory of New Energy Devices and Interface Science, School of Chemistry and Materials Science, Nanjing University of Information Science and Technology, Nanjing, 210044 People’s Republic of China; 2 Guangdong-Hong Kong-Macau Joint Laboratory for Photonic-Thermal-Electrical Energy Materials and Devices, Institute of Applied Physics and Materials Engineering, University of Macau, Macau, 999078 People’s Republic of China; 3 Key Laboratory of Physics and Technology for Advanced Batteries (Ministry of Education), Jilin University, Changchun, 130012 People’s Republic of China; 4 Jiangsu Key Laboratory of Electrochemical Energy Storage Technologies, College of Material Science and Technology, Nanjing University of Aeronautics and Astronautics, Nanjing, 211106 People’s Republic of China

**Keywords:** Aqueous batteries, Manganese storage, Electrode materials, Electrolytes, Energy storage mechanism

## Abstract

Manganese exhibits remarkable chemical versatility, arising from its multiple valence states and diverse coordination environments. This unique redox flexibility underpins a rich spectrum of electrochemical processes, making manganese-based compounds central to the development of advanced energy storage systems. However, it also gives rise to intrinsic instability, involving disproportionation reactions, dissolution of Mn species, and irreversible structural evolution. An in-depth understanding of these coupled chemical–structural dynamics is essential to unlocking the full potential not only of Mn-based electrodes but, more importantly, of aqueous Mn-ion batteries (AMIBs). In this review, we critically summarize the recent progress of AMIBs, with an emphasis on the development and engineering strategies of electrodes and electrolytes. Finally, we propose future perspectives for constructing robust, energetic, and sustainable AMIBs.

## Highlights

- A systematic elucidation of the fundamental electrochemical principles governing aqueous manganese batteries, focusing on the reaction mechanisms of key redox couples (e.g., Mn/Mn^2+^/Mn^3+^/MnO_2_), carrier characteristics (such as ionic radius and solvation effects), and their underlying impact on battery performance.
- A comprehensive and focused overview of recent advances in electrodes and electrolytes, including key modification strategies—pre-intercalation, coating, and interface regulation on electrode materials and optimizing electrolyte components (e.g., pH, additives) to suppress side reactions and widen the electrochemical window.
- A systematic analysis of the core challenges hindering the practical application, such as irreversible deposition/dissolution and electrode structural degradation. The future research directions were proposed, including electrode/electrolyte optimization strategies, characterization methods, and AI-assisted approaches, etc.

## Graphical abstract

**Figure:**
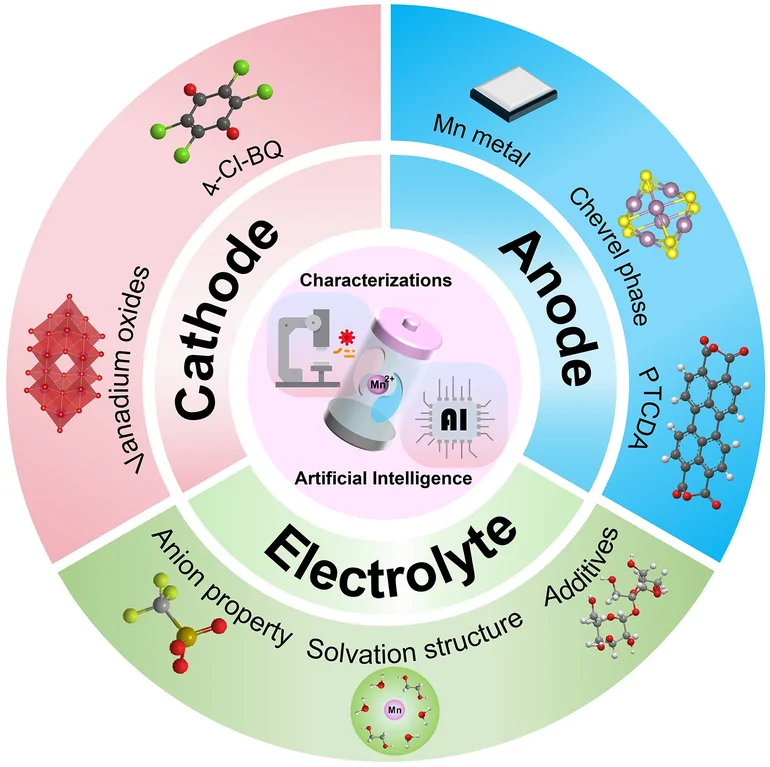

## Introduction

Manganese (Mn) has long been considered one of the most significant elements in the electrochemical energy storage (EES) field due to its natural abundance, low cost, environmental benignity, and rich multivalent redox chemistry [1, 2]. Leveraging accessible Mn(II/III/IV) couples, in particular MnO_2_/Mn^2+^ [3, 4] and Mn^3+^/Mn^2+^ [5, 6], together with Mn’s crystal–chemical diversity (e.g., *α*/*β*/*γ*/δ-MnO_2_ [7–10], layered and spinel Mn oxides [11, 12]), manganese compounds enable proton-coupled and cation-insertion reactions, empowering manganese compounds to function as electro-active materials [13–16] and electrolytes [17, 18] across various energy storage systems. Representative deployments span alkaline batteries [11, 19, 20], supercapacitors [21], and more recently, aqueous zinc-ion batteries (ZIBs) [22, 23]. Beyond these Mn couples, the MnO_4_^2−^/MnO_4_^−^ redox couple has been reported to show excellent reversibility in strong alkaline for redox flow batteries. The theoretical voltage can further reach 2.8 V in an acid-alkaline decoupling system. However, such a system suffers from poor reversibility, thus behaving like a primary cell rather than rechargeable cell [24]. Besides, manganese salts have also been used as additives in electrolytes to stabilize electrode interfaces or to regulate proton activity [25]. Taken together, this breadth of solid- and liquid-phase utility, rooted in variable valence, tunable frameworks, and aqueous compatibility, underscores Mn’s foundational role in safe and sustainable energy storage technologies and naturally motivates its role evolution from a supportive electrode constituent to an active ionic charge carrier.

**Fig. 1:**
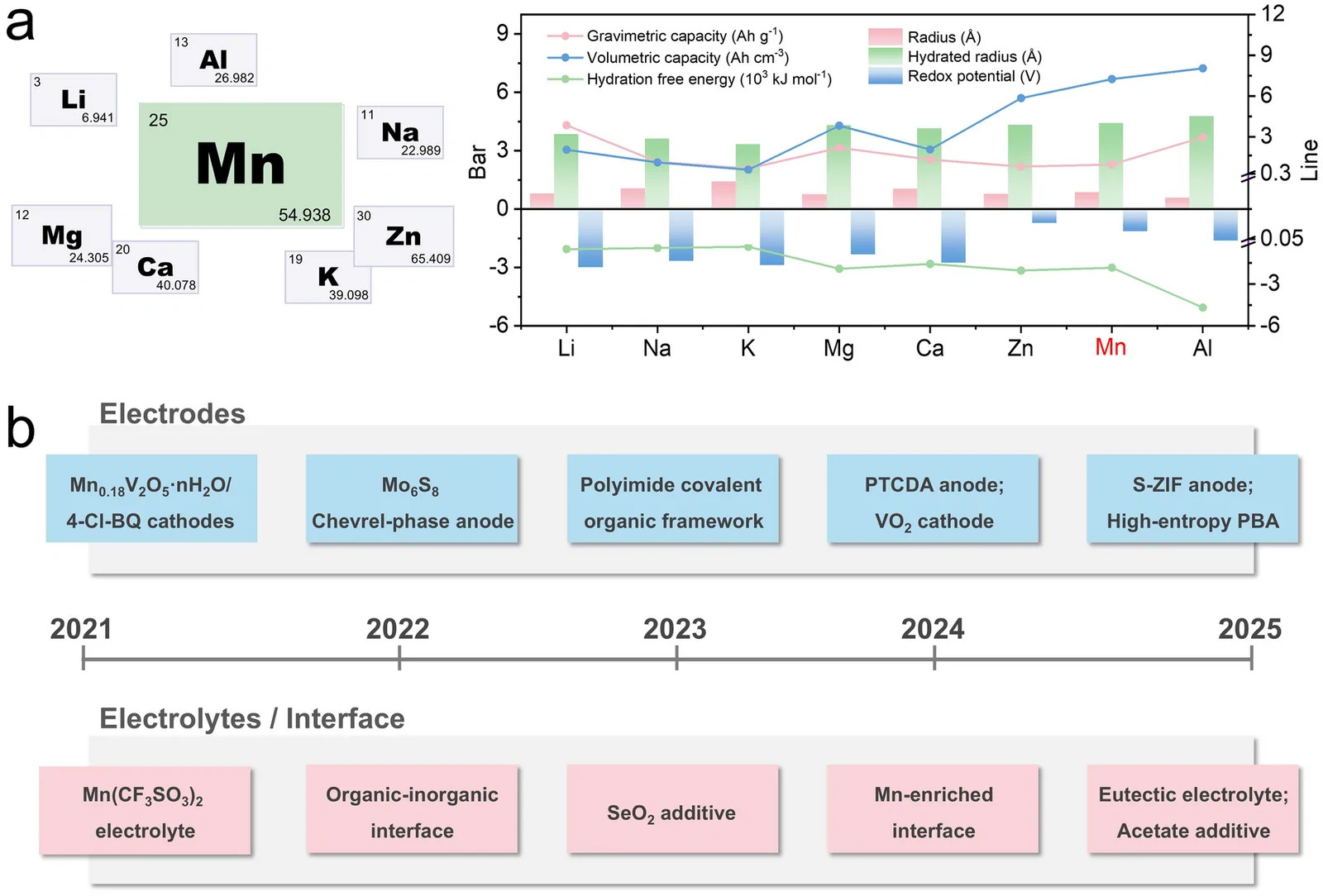
**a** Comparison of the properties of different metal elements. **b** Timeline of recent developments of AMIBs

However, the application of manganese as a primary charge carrier—in the form of Mn^2+^—is a relatively recent development. The short history of AMIBs can be attributed to the intrinsic challenges associated with Mn^2+^ transport and intercalation. Mn^2+^ has a large hydrated ionic radius (Fig. 1a) and forms a stable [Mn(H_2_O)_6_]^2+^ solvation shell. Its strong electrostatic interaction with host frameworks also leads to sluggish diffusion kinetics and significant structural strain during cycling [1]. These factors often result in limited rate capability [26] and rapid capacity decay [27]. Moreover, the dissolution issue of Mn compounds, especially MnO_2_ cathode [28], often results in continuous manganese loss and parasitic reactions associated with disproportionation reactions [29] and Jahn–Teller (J–T) distortion [30, 31], which further hinder long-term stability. These issues fundamentally limited the early exploration of AMIBs until Niu’s group pioneered the first aqueous manganese-ion battery in 2021 (Fig. 1b), demonstrating reversible Mn^2+^ intercalation into layered metal oxides [32]. Since then, numerous studies have explored the electrochemical behavior of Mn^2+^, revealing highly condition-dependent mechanisms [33–37]. Despite these advances, the fundamental understanding of Mn-ion chemistry remains incomplete, and overcoming its intrinsic limitations continues to be a central research focus.

**Fig. 2:**
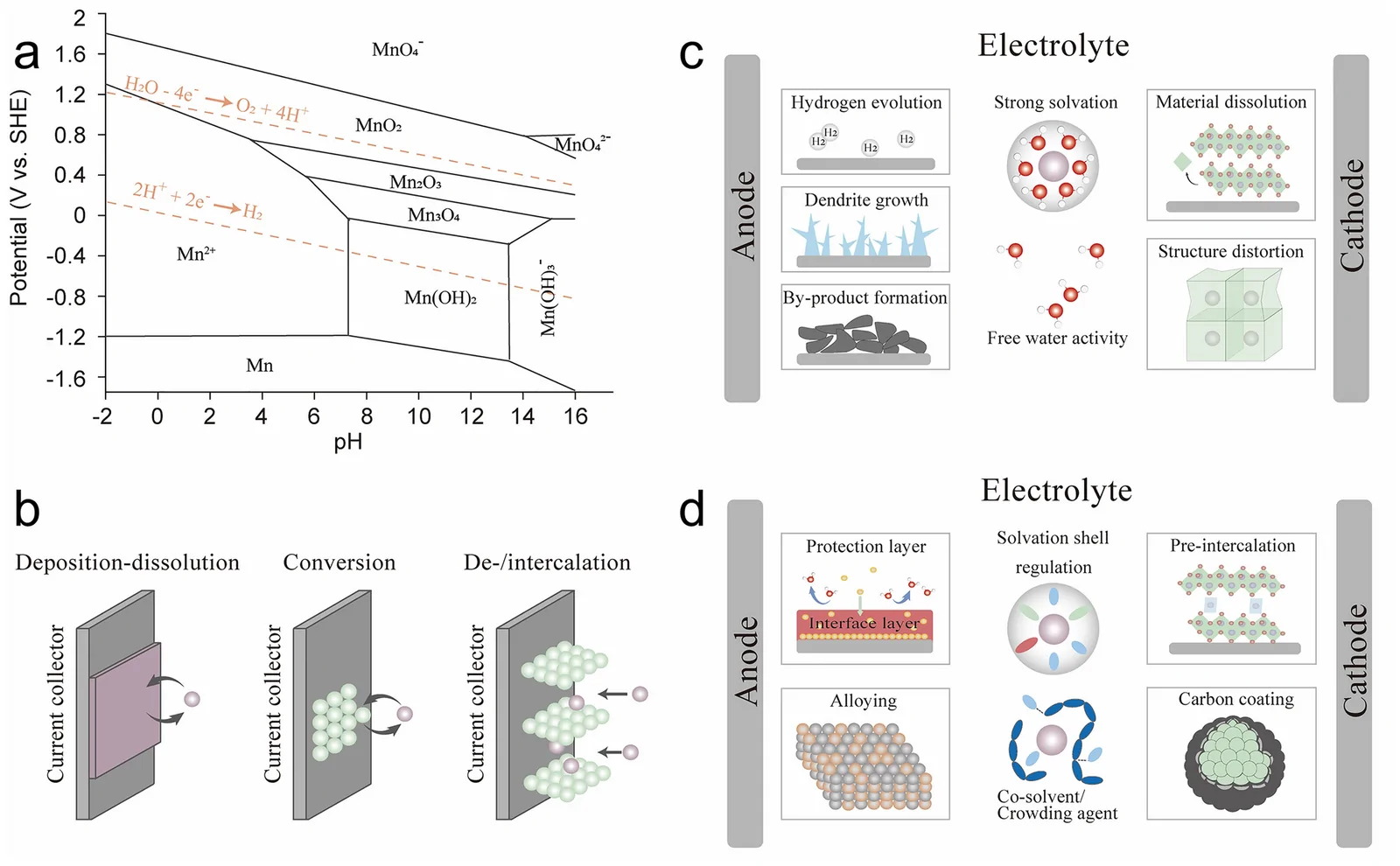
**a** Mn Pourbaix diagram. **b** Key storage mechanisms of electrode materials, including deposition–dissolution reaction, conversion reaction, and intercalation/de-intercalation reaction. **c** Central challenges of electrodes and electrolytes for AMIBs, and **d** representative strategies employed for AMIBs

In fact, many of the aforementioned challenges originate from the intrinsic electrochemical characteristics of manganese in aqueous media. The Mn/Mn^2+^/Mn^3+^/MnO_2_ redox system is much dependent on coordination chemistry and electronic structure. In contrast to Zn^2+^ and Mg^2+^ systems, which typically involve more stable divalent redox couples and relatively well-defined deposition or intercalation behaviors, manganese-based systems are uniquely complicated by the instability of intermediate valence states and the strong coupling between solvation, coordination, and interfacial chemistry. In particular, Mn^3+^ is thermodynamically unstable in aqueous environments and typically exists only as a short-lived intermediate. Owing to its high-spin d^4^ electronic configuration, Mn^3+^ exhibits strong Jahn–Teller activity, which induces pronounced distortion of Mn–O coordination units and renders local structures susceptible to reorganization during redox processes. In addition, Mn^2+^ ions possess a strongly hydrated solvation shell in aqueous electrolytes. Desolvation at the electrode interface is often slow. This feature frequently couples solid-state reactions with solution-mediated processes, especially during pH relevant process of Mn^2+^/MnO_2_ formation and dissolution (seen from Fig. 2a). These intrinsic physicochemical characteristics do not prescribe specific electrode or electrolyte design strategies, but rather define the fundamental electrochemical boundaries within which diverse manganese-based materials and reaction pathways operate in aqueous systems.

Against this backdrop, quantitative advantages distinguish Mn^2+^ from peer ions. The hydration free energy of Mn^2+^ (−1760 kJ mol^−1^) is slightly higher than that of Zn^2+^ (−1955 kJ mol^−1^) and Mg^2+^ (−1830 kJ mol^−1^), and is greatly higher than Al^3+^ (−4525 kJ mol^−1^) [38]. Desolvation barriers are therefore lower, benefiting interfacial charge transfer and in-host migration. Its redox potential of − 1.18 V vs. standard hydrogen potential (SHE) makes it ideal for constructing high-voltage aqueous systems in principle [39], surpassing the typical 2.0 V threshold seen in zinc-based batteries [40]. This provides the opportunity for high-energy–density batteries without compromising safety. Additionally, redox versatility of Mn enables multiple electrochemical pathways, including Mn deposition/stripping [37, 41–43], Mn^2+^/MnO_2_ deposition/dissolution [3, 24, 25], and liquid–liquid Mn^2+^/Mn^3+^ reaction [44–46]. The multivalent nature also allows for multiple electron transfer, offering high theoretical capacity (7,250 mAh cm^−3^ volumetric capacity and 976 mAh g^−1^ gravimetric capacity [32], Fig. 1a) and making it a promising candidate for energy-dense batteries. It is worth noting that these advantages are tightly coupled to the major obstacles faced by AMIBs. The low discharge potential of Mn metal inevitably exceeds the thermodynamic limits of water [47], intensifying parasitic hydrogen evolution and electrolyte decomposition. Meanwhile, the multivalent redox states of Mn may lead to side reactions and structural instability of cathode materials that reduce overall battery performance [48]. While Mn-based systems offer high voltage and energy density, their performance is inevitably constrained by thermodynamic and kinetic instabilities inherent to water-based chemistries. Thus, integrated design approach is needed to overcome these intertwined challenges.

Since the pioneering works have been put forward (related works are listed in Table 1), in this review, we provide a comprehensive overview on recent advances and key elements of AMIBs, briefly illustrated in Fig. 1b. In practical cell design, the Mn Pourbaix (pH–potential) diagram (Fig. 2a) can serve as a convenient reference to guide pH and potential windows and to anticipate possible reaction pathways. Regarding electrode design, we discuss the relationships between the material structure and the electrochemical behavior and introduce the energy storage mechanisms (Fig. 2b). Specifically, Mn can participate through insertion/extraction within host frameworks or MnO_2_/Mn^2+^ deposition–dissolution conversion reactions on the cathode side, whereas on the anode side, it can operate via Mn^2+^/Mn plating-stripping or via intercalation process in non-metal anodes. On this basis, the challenges and optimization strategies of electrode materials are proposed (seen in Fig. 2c, d), providing inspiration for subsequent research. Regarding electrolyte modulation, we introduce the strategies used in AMIBs, such as adding additives/co-solvents and regulating solvation shell (Fig. 2c). Finally, considering the infancy stage and the existing challenges of AMIBs, we propose strategic perspectives, hoping to exert efforts to build advanced AMIBs.

**Table 1:** Summary of reported AMIBs

Electrode	Electrolyte	Capacity (mAh g^−1)^	Current density (A g^−1^)	Cycling stability	Refs
Mn||Mn_0.18_V_2_O_5_·nH_2_O	1 M Mn(CF_3_SO_3_)_2_	133.7	0.2	86.7% 200 cycles at 5.0 A g^−1^	[32]
AC||LiV_3_O_8_	3 M Mn(ClO_4_)_2_	280	0.1	85% 30,000 cycles at 6.0 A g^−1^	[49]
AC||Al_0.1_V_2_O_5_·1.5H_2_O	1 M Mn(CF_3_SO_3_)_2_	320.9	0.5	~ 100% 1000 cycles at 5 A g^−1^	[35]
AC||Na_1.25_V_3_O_8_	1 M Mn(ClO_4_)_2_	240	0.1	97% 10,000 cycles at 4.0 A g^−1^	[50]
AC||VO_2_	1 m Mn(CF_3_SO_3_)_2_ in 50 vol% EG	383	0.5	162 mAh g^−1^ 20,000 cycles at 5 A g^−1^	[51]
AC||Ag_0.33_V_2_O_5_	Saturated Mn(ClO_4_)_2_	261.9	0.1	69.1% 2000 cycles at 1.5 A g^−1^	[52]
AC||Ni_0.48_V_2_O_5_·0.90H_2_O	3 M MnSO_4_	216.1	0.05	~ 100% 3000 cycles at 2.0 A g^−1^	[53]
Mn||V_2_O_4.85_	Saturated MnCl_2_	212.6	0.1	89.5% 500 cycles at 0.1 A g^−1^	[54]
Mn||V_2_O_5_-CNT	1 M Mn(CF_3_SO_3_)_2_	300.5	0.5	96% 1000 cycles at 5.0 A g^−1^	[55]
Mn||4-Cl-BQ	1 M Mn(CF_3_SO_3_)_2_	~ 120	0.2	~ 100% 80 cycles at 1.0 A g^−1^	[32]
VO_2_||Mn-HEPBA	0.1 M MnSO_4_	117.9	0.1	82.8% 5000 cycles at 1.0 A g^−1^	[56]
AC||NaV_6_O_15_/C	3 M Mn(ClO_4_)	252.4	0.1	74% 6000 cycles at 3.0 A g^−1^	[57]
ZIF-S||MnO_2_	4 m MnSO_4_	1242	0.5	56.1% 130 cycles at 0.5 A g^−1^	[58]
Mn@MIL||V_2_O_5_	1 M MnCl_2_	270	0.5	300 mAh g^−1^ 110 cycles at 0.5 A g^−1^	[42]
MnP||Ag-V_2_O_5_	1 M Mn(CF_3_SO_3_)_2_	204	0.5	88.2% 7000 cycles at 3 A g^−1^	[59]
Mn||MnO_2_	1 M MnSO_4_- 1 M (NH_4_)_2_SO_4_- 0.02 g L^−1^ SeO_2_	900 Wh kg^−1^	/	300 cycles at10 mA cm^2^	[37]
Mn||V_2_O_5_	MnSO_4_-NaClO_4_-glycine- 50 wt% sucrose	180	0.5	~ 100% 200 cycles at 1.5 A g^−1^	[43]
PTCDA||Mn-HEPBA	0.1 M MnSO_4_	140	0.1	65.5% 1800 cycles at 2 A g^−1^	[60]
PTCDA||AC	3 M MnCl_2_	187	0.1	43.3% 250 cycles at 1.6 A g^−1^	[33]
PI-COF||MnO_2_	2.0 M MnSO_4_	120	0.2	92.9% 100 cycles at 1.0 A g^−1^	[61]
Mo_6_S_8_||NiHCF	Saturated MnCl_2_	93	0.5	> 96% 1500 cycles at 5 A g^−1^	[34]
Mn||AgVO	3 M Sodium alginate@Mn	~ 200	0.5	150 mAh g^−1^ 410 cycles at 1.5 A g^−1^	[62]
PTCDI||TCBQ	0.5 M MnSO_4_- 0.5 M MnAc_2_	100	1.0	90.5% 500 cycles at 1.0 A g^−1^	[63]
PTCDI||NiHCF	Mn(ClO_4_)_2_·6H_2_O-acetamide	98.4	0.2	95.6% 1200 cycles at 1.0 A g^−1^	[64]

AC = active carbon; CNT = carbon nanotube; 4-Cl-BQ = TCBQ = Tetrachloro-1,4-benzoquinone; Mn-HEPBA = Na_1.19_Mn_0.4_Fe_0.15_Ni_0.15_Co_0.15_Cu_0.15_[Fe(CN)_6_]_0.81_·_□0.19_·1.6H_2_O; MIL = Mn-enriched interfacial layer; PTCDA = 3,4,9,10-Perylenetetracarboxylic dianhydride; PI-COF = Polyimide covalent organic framework; PTCDI = Perylene-3,4,9,10-tetracarboxylic diimide

## Cathode Materials for AMIBs

Cathode materials play a key role in aqueous batteries, fundamentally dictating their performance, cost, and practical viability. Developing high-performance cathode materials in an aqueous system demands meticulous design. Ideal candidates are supposed to exhibit high redox potentials to maximize voltage within the electrolyte’s stability window, high specific capacity to store and release charge carriers, and excellent kinetics to facilitate fast ion insertion/extraction for high power delivery. Superior structural stability is essential to maintain integrity over charge/discharge cycles within the aqueous medium, coupled with minimal dissolution to prevent active material loss into the electrolyte. Consequently, the selection and design of cathode materials are paramount for advanced AMIBs. To date, most reported cathodes are based on vanadium oxides and organic materials, and representative advances are outlined as follows.

### Vanadium-Based Oxides

**Fig. 3:**
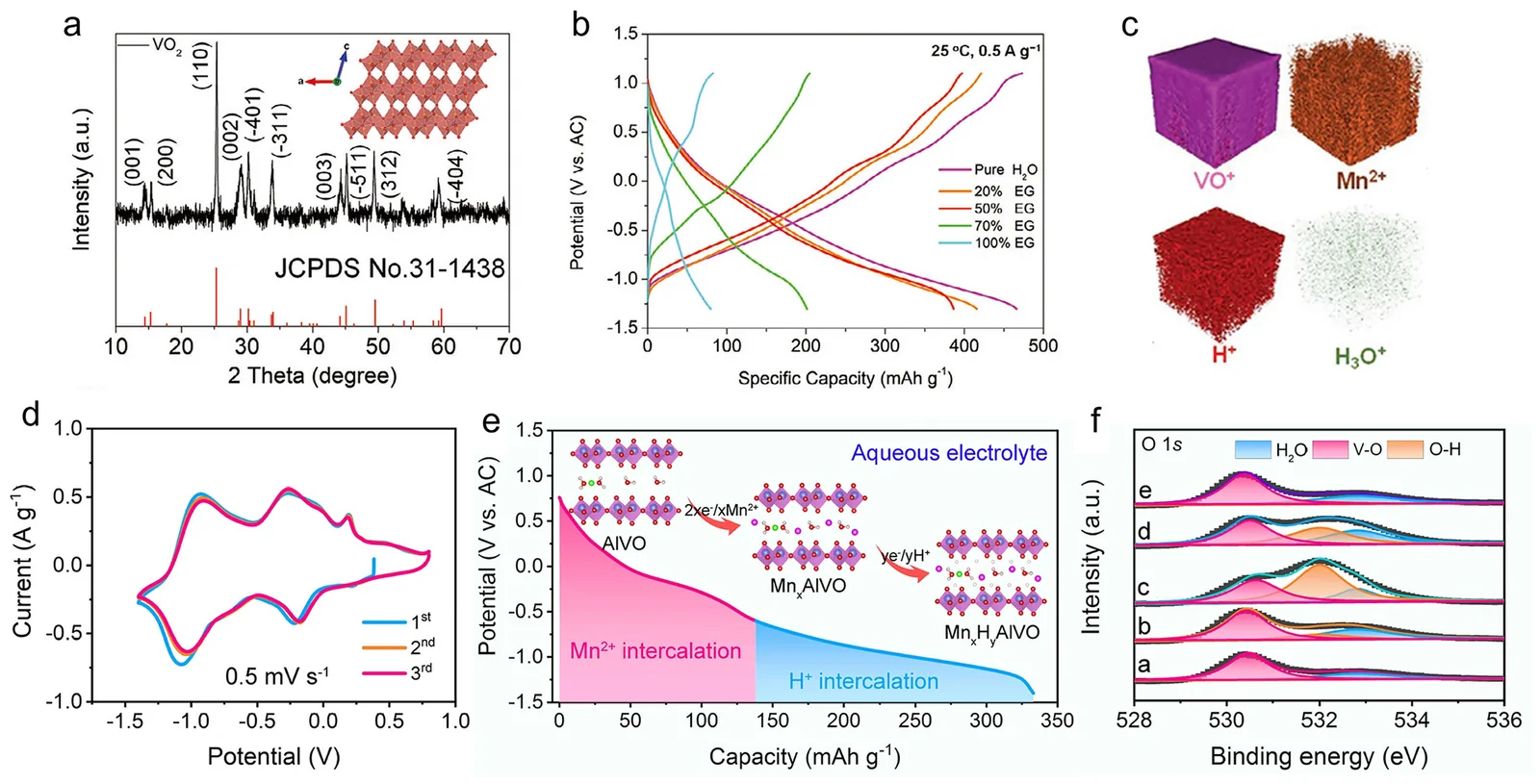
**a** X-ray diffraction (XRD) and **b** galvanostatic charge–discharge (GCD) curves of VO_2_ in various electrolytes at 0.5 A g^−1^. **c** Energy storage mechanism of Mn^2+^/proton co-insertion revealed by 3D TOF–SIMS images. **a–c** Reproduced with permission [51]. **d** CV curves at 0.5 mV s^−1^ of the AlVO. **e** Schematic diagram of the discharging mechanism of the AlVO, indicating a consequent intercalation process of Mn^2+^ and H.^+^. **f** Ex situ X-ray photoelectron spectroscopy (XPS) of O 1*s* at selected states, demonstrating the formation of Mn(OH)_2_. **d**, **f** Reproduced with permission [35]

As a transition metal element, vanadium has multiple valance states varying from + 2 to + 5, which enables multielectron transfer and high-capacity potential [65]. In widely investigated aqueous ZIBs, vanadium dioxide (VO_2_) as cathode material attracts a large portion of attention due to its high-capacity and high-rate tolerance property [66–69]. Such advantages are also validated in AMIBs by Mai’s group [51]. They synthesized monoclinic tunnel-typed VO_2_ nanorods (Fig. 3a), which exhibited high capacities both in pure aqueous electrolyte and ethylene glycol (EG) mixed electrolyte (Fig. 3b). Time-of-flight secondary ion mass spectrometry (TOF–SIMS), shown in Fig. 3c**,** revealed a co-insertion phenomenon of non-hydrated protons, leading to the elevation of OH^−^ concentration and reversible formation of Mn_m_(Otf)_n_(OH)_2 m-n_ by-product. In situ pH measurements and spectroscopic analyses further confirmed that the formed by-product could effectively act as a pH buffer and accelerated charge transfer kinetics. Density functional theory (DFT) and molecular dynamics (MD) simulations revealed that the introduction of EG altered the hydrogen-bond network and suppressed fast Grotthuss proton conduction (proton jumps between conducting mediums accompanied with a rotational reorientation motion and low activation energy below 0.4 eV [70]), thereby mitigating uncontrolled proton migration.

Layered vanadium-based oxides cannot be neglected in rechargeable aqueous batteries (RABs) as the large interspacing generally exhibits excellent metal ion accommodation ability [71–73]. This makes it rational to surmise the potential use in hosting Mn^2+^ ions, which was first proved by Niu’s group [32]. By introducing Mn^2+^ ions into the interlayer of V_2_O_5_, a hydrated vanadium oxide Mn_0.18_V_2_O_5_·nH_2_O (MVO) cathode was synthesized and delivered 133.7 mAh g^−1^ capacity at 0.2 A g^−1^. In another work, Niu’s group pre-intercalated Al^3+^ into the interlayer of V_2_O_5_ and obtained Al_0.1_V_2_O_5_·1.5H_2_O (AlVO) cathode material [35]. Unlike MVO cathode, the synthesized AlVO enabled Mn^2+^ /protons two-step consequent insertion (indicated by two reduction peaks shown in Fig. 3d), and delivered a higher capacity of 320.9 mAh g^−1^ at 0.5 A g^−1^ (Fig. 3e**)**. Meanwhile, Fig. 3e showed that the capacities associated with proton insertion and Mn^2+^ insertion are comparable, indicating that the two charge carriers contribute similarly to the overall capacity, whereas Lee et al. demonstrated the primary role of Mn^2+^ in V_2_O_4.85_ cathode [54]. While proton involvement can boost apparent capacity, the generation of Mn(OH)_2_, detected by ex situ XPS at discharged state (Fig. 3f), also suggests that local pH shifts may trigger secondary phases that could compromise reversibility. The distinct storage mechanism is described briefly as below:

1 $$y\mathrm{H}_{2}\mathrm{O}\leftrightarrow y\mathrm{H}^{+}+y\mathrm{OH}^{-}$$

2 $$\mathrm{AlVO}+x\mathrm{Mn}^{2+}+y\mathrm{H}^{+}+\left(2x+y\right){e}^{-}\leftrightarrow {Mn}_{x}{H}_{y}\mathrm{AlVO}$$

3 $${y\mathrm{OH}}^{-}+\frac{1}{2}y\mathrm{Mn}^{2+}\leftrightarrow \frac{1}{2}y{\mathrm{Mn}\left(\mathrm{OH}\right)}_{2}$$

Similarly, proton co-insertion phenomenon and generation of Mn(OH)_2_ by-product were also reported by Lee et al. [52]. The synthesized Ag_0.33_V_2_O_5_ (AgVO) cathode material exhibited two distinct configuration forms with linear chains of VO_6_ octahedra and VO_5_ square pyramids. The unit cell contained two identical crystallographic sites with only one site active. The proton co-insertion process was evidenced by complex redox peaks in cyclic voltammetry (CV) profiles, and a three-step discharge mechanism, including (1) generation of Mn hydrated by-products; (2) formation of Mn(OH)_2_ and (3) precipitation of Ag^+^ ions in AgVO on the ζ-V_2_O_5_ surface, was identified. Migration pathway simulation revealed that Mn^2+^ ions preferred surface redox reactions and ion hopping while protons diffused along 1D b-axis and across c-axis (prior over Mn insertion that leads to side reactions). Likewise, Soundharrajan et al. observed the in situ formation of MnClO_4_·xH_2_On(OH)_y_ by-product in a carbon-coated NaV_6_O_15_ (NVO/C) framework. And the authors proposed that this compound could act as a promoter to facilitate H^+^ (de)insertion [57]. The frequently observed proton participation and associated by-product chemistry indicate that proton co-insertion is a broadly relevant feature rather than an isolated case.

In summary, vanadium-based oxides offer high capacities and benign rate performance due to their multivalent redox activity and diverse structural forms. Emerging strategies including pre-intercalation, and carbon-coated modifications, etc., have shown promise in stabilizing frameworks and improving ion transport. Concurrently, proton involvement is frequently observed in these systems, which requires careful regulation and interpretation. While proton involvement can provide synergistic benefits to capacity and kinetics, it also adds complexity to reaction pathways and may induce by-product formation. These works suggest that it would be beneficial for proton participation when local pH variations are reversibly regulated. Nevertheless, cycling stability remains a critical challenge, and future research should focus on figuring out the respective roles of Mn^2+^ and protons, while integrating material and electrolyte engineering to achieve long-term performance.

### Prussian Blue Analogs

Prussian blue analogs (PBAs) have been regarded as one of the most promising cathode materials due to their open framework, large interstitial channels, low cost, and facile synthesis [74–76]. Their robust structure enables rapid ion insertion/extraction and high structural stability during cycling. PBAs have been extensively studied and have proven their importance in battery systems [77–80]. However, their application in AMIBs remains largely unexplored. While a few recent studies have employed PBAs as Mn^2+^-hosting cathodes, these works primarily focus on initial electrochemical performance metrics [34, 58, 64]. A comprehensive mechanistic understanding of how Mn^2+^ is stored within the PBA lattice is still lacking, which makes it difficult to assess the intrinsic advantages of PBAs compared with other hosts.

**Fig. 4:**
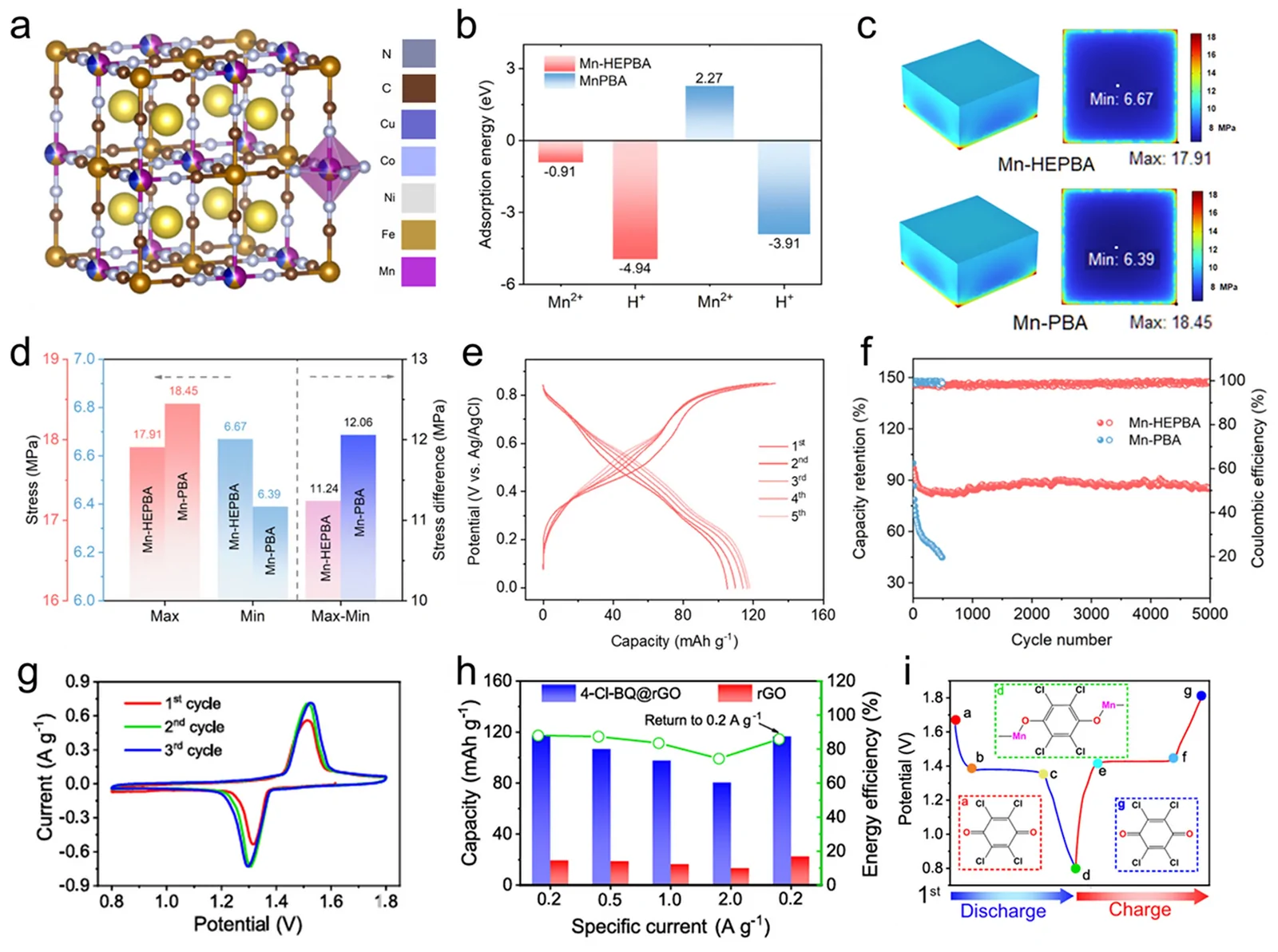
**a** Schematic illustration of Mn-HEPBA structure. **b** Adsorption energy of Mn^2+^ and H^+^ on Mn-HEPBA and Mn-PBA. **c** Three-dimensional von Mises stress distributions and corresponding ground stress distributions. **d** The maximum/minimum von Mises stress and the stress difference. Mn-HEPBA shows reduced maximum von Mises stress, indicating weakened stress concentration. **e** GCD profiles of Mn-HEPBA at 0.1 A g^−1^, and **f** cycling performance at 1.0 A g^−1^. **a**–**f** Reproduced with permission [56]. **g** CV curves at 0.2 mV s^−1^. **h** Capacities of rGO and 4-Cl-BQ@rGO composites at different specific currents. **i** The first GCD curve with the schematic diagrams of 4-Cl-BQ molecules at different discharge/charge states. **g–i** Reproduced with permission [32]

Recently, Dong’s group reported a Mn-based high-entropy PBA (Mn-HEPBA) cathode for Mn^2+^ storage [56]. The material, with five transition metals (Mn, Fe, Ni, Co, Cu) occupying the nitrogen-coordinated sites (structure illustration is shown in Fig. 4a), fundamentally alters ion storage behavior: while conventional Mn-PBA relied on Mn^2+^/H^+^ co-insertion in MnSO_4_ electrolyte, Mn-HEPBA enabled dominant Mn^2+^-selective storage with minimal proton interference. In Fig. 4b, DFT calculations revealed spontaneous Mn^2+^ adsorption on Mn-HEPBA (− 0.91 eV) versus non-spontaneous adsorption on Mn-PBA (+ 2.27 eV), explaining the ion selectivity. In addition, finite element simulations (Fig. 4c) showed smaller stress difference of Mn-HEPBA between the maximum and minimum (Fig. 4d), an evident reduction in von Mises stress gradient compared to Mn-PBA. This highlights the potential of high-entropy engineering as a novel approach to optimize Mn^2+^ affinity and to suppress parasitic reactions by dispersing local stress, mitigating framework distortion during ion insertion. Consequently, Mn-HEPBA delivered a high capacity of 117.9 mAh g^−1^ at 0.1 A g^−1^ (Fig. 4e) and retained 64 mAh g^−1^ (82.8% capacity retention) after 5000 cycles at 1 A g^−1^ (Fig. 4f). Ex situ characterizations elucidated a reversible cubic-to-monoclinic phase transition and verified the redox activity at Mn, Fe, Co, and Cu sites. Moreover, Mn dissolution was suppressed, conforming with the cycling stability. These results clearly demonstrate that PBAs, when rationally engineered, can achieve both decent capacity and outstanding long-term cycling stability, though further studies are needed to clarify the detailed Mn^2+^ transport pathways inside the open framework.

In summary, PBAs stand out as structurally open and chemically versatile cathodes for AMIBs. Their capacity is generally lower than that of vanadium-based oxides, but their desirable cycling stability and ease of synthesis make them highly attractive for practical applications. The development of high-entropy PBAs exemplifies how novel material design strategies can overcome limitations of conventional PBAs by tuning mechanical robustness. Although many works have already reported PBAs as AMIB cathodes, systematic mechanistic studies are still scarce, and future research should focus on clarifying Mn^2+^ storage pathways and establishing long-term stability under realistic conditions.

### Organic Cathode Materials

Organic materials have been considered as another one of the most promising candidates for renewable energy storage. As widely recognized, organic molecules possess diverse structures with tunable functional groups, and thus can be applied in different ESSs [81, 82]. Based on the conversion reaction of functional groups, organic molecules are capable of accepting ions with different sizes, while metal oxides usually have specific requirements for inserted ions [83]. Meanwhile, organic compounds are generally composed of C, H, O, N, and S elements, which are abundant in nature, thus making them cost effective [84]. The recyclable merit of organic compounds also makes them more environmentally friendly and favored [84]. These advantages make organic electrodes attractive alternatives to inorganic hosts, particularly when sustainability, structural tunability, and environmental considerations are emphasized.

Among various organics, compounds with conjugated carbonyl groups are widely adopted as conversion-type electrode materials [85]. Such a conversion mechanism endows them with the capability to accommodate large ions, therefore competitive candidates for AMIBs. For instance, tetrachloro-1,4-benzoquinone (4-Cl-BQ) has been validated as an effective choice [32]. To relieve the dissolution issue and improve conductivity, 4-Cl-BQ was incorporated into reduced graphene oxide (rGO) foams (the characteristic CV curves are presented in Fig. 4g) and consequently delivered 80.6 mAh g^−1^ capacity at 2 A g^−1^ (68.5% retention of that at 0.2 A g^−1^), Fig. 4h. Moreover, the introduction of rGO also benefited the cyclicity that the capacity could maintain 75.6 mAh g^−1^ after 80 cycles (the initial capacity was 71.9 mAh g^−1^ at 1 A g^−1^). Ex situ XRD/Fourier transform infrared spectroscopy (FTIR) measurements were also conducted to probe the evolution process. A conversion reaction from C = O to C − O^−^ happened when Mn^2+^ ions inserted, and were reversibly removed during the charging process, indicating the mechanism an enolization redox reaction (Fig. 4i). DFT further uncovered the coordination site at fully discharged state where Mn^2+^ ions’ uptake took place along the b axis between adjacent 4-Cl-BQ molecules, and the resulting product enabled a more stable crystal structure. This example highlights how rational engineering approaches, such as hybridization with conductive carbon networks, can mitigate dissolution and enhance conductivity, thereby improving both reaction kinetics and cycling stability.

In brief, organic cathodes provide a unique balance between sustainability and structural flexibility. Their relatively lower capacity compared with vanadium-based oxides is counterbalanced by abundant elemental resources and environmental friendliness. However, their practical application still faces challenges including poor conductivity, dissolution into electrolytes, and limited cycling stability. Engineering strategies such as composite formation with conductive scaffolds, molecular structure tuning, and incorporation of stabilizing frameworks have already shown positive effects on enhancing battery performance. At present, reports on organic cathodes for Mn^2+^ storage are very limited, indicating that this field is still in its infancy and requires systematic exploration. To further advance this class of materials, more detailed mechanistic studies and long-term cycling evaluations are required, so that the advantages of organics can be effectively harnessed without compromising performance in AMIBs.

Overall, vanadium-based oxides, PBAs, and organic materials represent three distinct categories of cathodes for AMIBs, each exhibiting distinct advantages and limitations in terms of capacity, kinetics, and cycling stability (briefly summarized in Table 2). While diverse structural engineering strategies have enabled remarkable performance improvements, their effectiveness is ultimately governed by the intrinsic structural and electronic characteristics. Current studies primarily reveal that Mn^2^⁺ storage in these hosts is frequently accompanied by proton-coupled reactions, especially for vanadium oxides, which can boost apparent capacity/kinetics but also increase pathway complexity. This may arise from their rich valence states and coordination environments. Even though detailed proton storage mechanism is not explored yet, relevant studies in AZIBs demonstrate that band structure and valance state of vanadium are highly linked proton involvement [86]. By contrast, PBAs feature rigid cyanide-bridged frameworks and complex d-electron configurations [87]. These characteristics may impose kinetic and electronic constraints on proton-coupled redox processes. Likewise, proton storage in organic materials is a complex process, often accompanied by Coulombic repulsion [88].

**Table 2:** Comparisons between different cathode materials

Cathode	Property	Performance
Vanadium oxides	Rich valance state	High capacity, good rate performance
PBAs	Complex d-electron structure	Moderate capacity, good cycling stability
Organics	C=O conjugated frameworks	Moderate capacity

Besides, although direct experimental evidence for Mn^3+^-induced Jahn–Teller distortion in most reported cathodes remains limited, especially for manganese-based electrodes, the intrinsic electronic instability of high-valence manganese species may still impose fundamental constraints on long-term structural integrity under repeated cycling. To mitigate such Mn^3+^-associated structural instability, transition metal substitution is an effective strategy to tune the electronic structure and weaken the driving force for Jahn–Teller distortion. This approach is particularly applicable to Mn-based oxides and Mn-based PBAs, where partial replacement of Mn sites can help suppress lattice deformation and improve long-term cycling stability [89]. In addition, surface coating offers another strategy by regulating the spin energy level of the Mn 3d-electrons, thereby suppressing Mn dissolution and distortion [90]. Meanwhile, the strong solvation of Mn^2+^ and the sensitivity of reaction pathways to local chemical environments further complicate performance optimization. Therefore, future cathode development should integrate systematic mechanistic investigations with rational material and electrolyte design to clarify dominant degradation pathways and enable durable AMIB systems.

Beyond the cathodes discussed above, the Mn^2+^/MnO_2_ dissolution–deposition reaction is another commonly adopted cathode chemistry in aqueous Mn-based full cells. Its reversibility and stability are highly sensitive to local electrolyte chemistry (notably pH evolution). Although many AMIBs full-cell configurations rely on Mn^2+^/MnO_2_ processes [37, 58, 61, 91], targeted mechanistic investigations of this reaction pathway in the AMIBs context remain relatively limited, which hinders a comprehensive understanding of its potential impact on system-level. Evaluating and optimizing this chemistry requires explicit attention to electrolyte regulation and crystal structure. This perspective complements the host-insertion cathode discussion above and underscores the importance of electrolyte control in aqueous Mn-based systems.

## Anode Materials for AMIBs

Another critical deficiency for AMIBs is the lack of anode materials. The performance of the anode strongly affects working voltage and energy density. Crucially, in most metal batteries, anode property is the key indicator for the overall safety of the system. The selection and design of high-performance and stable anode materials are therefore indispensable for realizing practical aqueous battery. The development of aqueous batteries is motivated by their enhanced safety profile and potential sustainability benefits compared to conventional organic electrolyte-based systems [92]. However, the aqueous environment presents distinct and often more severe challenges for anode materials than for cathodes, especially for Mn metal anode that operates at low potentials. Thus, the anode design for AMIBs should be carefully considered to achieve decent battery performance. However, anode materials are also in the infancy. To date, only a few studies offer effective attempts on the anode side.

### Mn Metal Anode

Due to the different electrochemical behaviors, Mn metal and intercalation-type materials are utilized as anode materials. Niu’s group first used Mn metal anode via coating a slurry of Mn metal powder onto the carbon felt surface [32]. However, the research focuses on the cathode, and the mechanism of this anode is not given in detail. In fact, the direct usage of Mn metal anode has to encounter the following issues of severe hydrogen evolution reaction (HER), corrosion, and dendrite growth. Unlike the zinc anode with the redox potential at ~ -0.76 V (vs. SHE), HER is more pronounced during the Mn deposition/dissolution process. The continuous generation of bubbles on the anode surface will hinder the intimate contact between Mn^2+^ ions and the metal surface, leading to an unsatisfactory Coulombic efficiency and poor cyclicity. These limitations highlight why practical use of Mn metal anodes is highly dependent on strategies that can suppress HER and stabilize deposition.

**Fig. 5:**
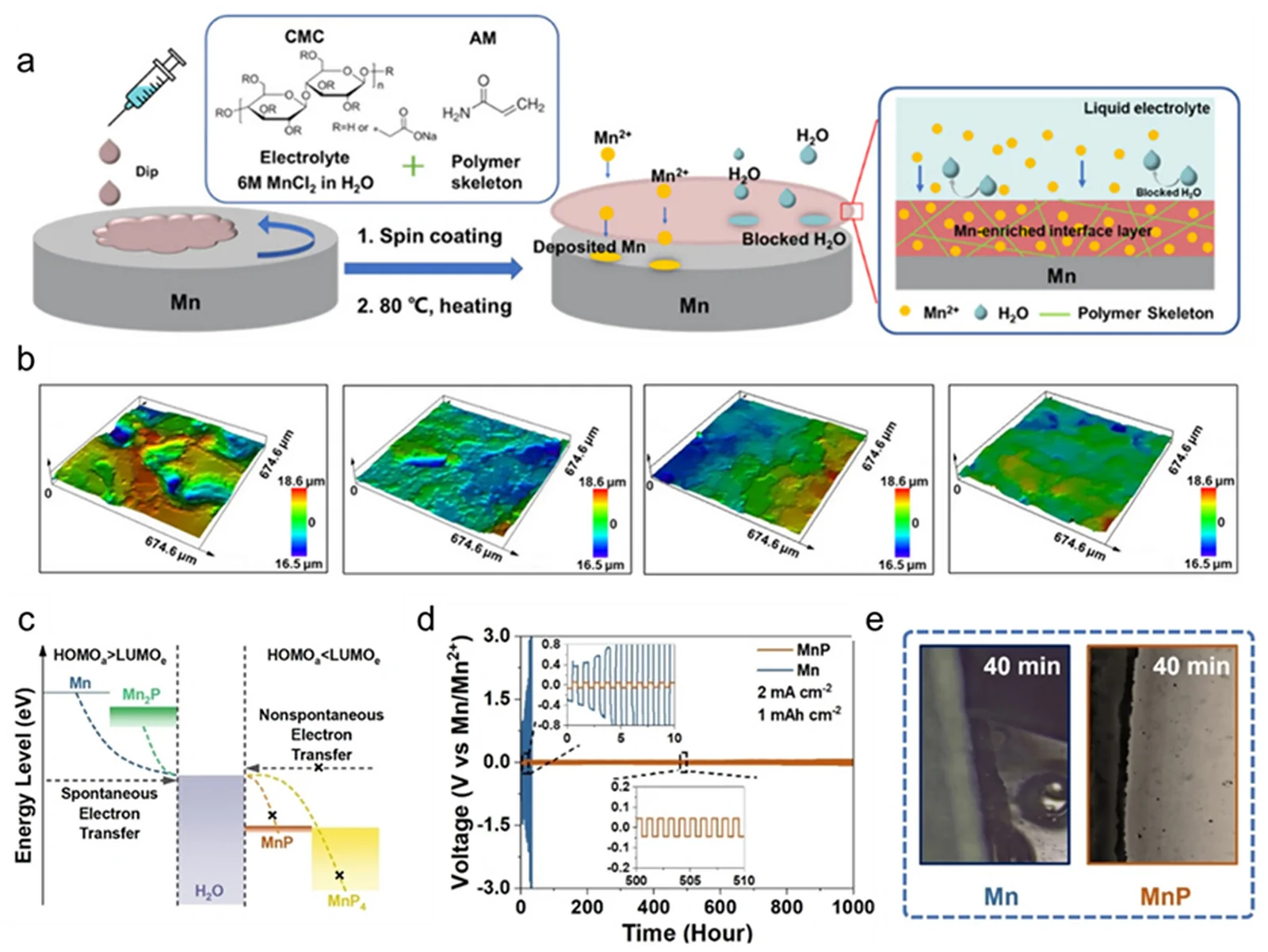
**a** Schematic illustration of the preparation process for the Mn@MIL, **b** 3D optical surface morphology images of different interfacial layers. From left to right are bare Mn||1M/6M MnCl_2_||Mn, and 1M/6M Mn@MIL||1M MnCl_2_||1M/6M Mn@MIL. 6M Mn@MIL cell exhibited the most uniform Mn deposition. **a, b** Reproduced with permission [42]. **c** LUMO energy level of the Mn and MnP alloys. As phosphorus content increases during the alloying process, the HOMO energy level decreases to reduce the propensity for HER. **d** Cyclic stability of symmetric cells at 2 mA cm^−2^/1 mAh cm^−2^, and **e** In situ photographs of the Mn and MnP anodes (side view). **c–e** Reproduced with permission [59]

Hou et al. proposed a Mn-enriched interfacial layer (1 M/6 M Mn@MIL) to isolate water clusters from the anode surface (illustrated in Fig. 5a) [42]. The Mn content in the interfacial layer was adjusted by dissolving 1 M or 6 M MnCl_2_ salts into acrylamide, *N, N*'-methylenebisacrylamide, and ammonium persulfate mixed solution. After acrylamide polymerization, the obtained coating layer not only acted as a physical barrier to suppress HER but facilitated Mn^2+^ diffusion to realize even deposition that prevented dendrite formation. The corresponding 3D optical surface topography of Mn deposition displayed in Fig. 5b exhibited the most uniform distribution of 6 M Mn@MIL in 1 M MnCl_2_ electrolyte, demonstrating the effectiveness of the surface modification. Meanwhile, the distinction between 1 and 6 M Mn@MIL suggested the acceleration of Mn^2+^ migration across the interfacial Helmholtz layer enabled by the Mn-enriched coating layer. Zhi’s group constructed an inorganic–organic interface to mitigate HER [43]. The inorganic interface was fabricated using Sn with chemical and electrochemical inertness to suppress water decomposition at the Mn–electrolyte interface and build a bubble-free environment. The ester-based organic interface was in situ formed via an electro-triggered esterification reaction. Cl· radicals from NaClO_4_ reduction reacted with hydrolyzed sucrose to form acyl chlorides. These then react with sucrose hydroxyl groups, forming a dense ester-based interface. This dense organic protective layer further stabilized the Mn metal/electrolyte interface while ensuring the diffusion of Mn^2+^ ions. Consequently, 200-h stable plating/stripping was achieved. These interfacial engineering approaches show how modifying the electrode/electrolyte interface can effectively suppress side reactions and achieve smoother Mn deposition, offering an important pathway to improve anode reversibility and extend cycling life.

In addition to interfacial engineering, Du’s group designed a series of Mn-P alloy anodes to mitigate the HER issue [59]. It is proposed that the HER in AMIBs comes from the spontaneous reduction (chemical process) and the electrocatalytic splitting (electrochemical process) of water molecules, and the severe HER on Mn metal anode is primarily caused by the high highest occupied molecular orbital (HOMO) energy level of Mn. Thus, through alloying Mn and P, band structures were regulated, and the HOMO energy level was significantly lowered (Fig. 5c). Ultraviolet photoemission spectrum and DFT calculations revealed the enlargement of work function with the increase in phosphorus content, indicating a reduced tendency of electrons to escape and reduce water. Density of states (DOS) analysis further revealed that metallic Mn exhibited a localized band structure with strong coupling to H 1*s* orbitals, forming excessively strong Mn–H bonds that hinder hydrogen desorption. By contrast, Mn–P alloys with increasing phosphorus content showed broadened *sp* bands with weaker H 1*s* coupling, which reduces interfacial hydrogen bonding. Consistent with this, the calculated Gibbs free energy of HER shifted from − 0.43 eV for Mn to − 0.13 eV (Mn_2_P), + 0.24 eV (MnP), and + 0.31 eV (MnP_4_), confirming that alloying effectively attenuates hydrogen adsorption. As a result, MnP anode exhibited desirable electrochemical properties during alloying/dealloying process with stable cycling performance over 1000 h at 2 mA cm^−2^ (Fig. 5d) and 5 mA cm^−2^, and the spontaneous parasitic reactions were restricted, which was confirmed via in situ optical microscopy as no bubbles were observed on the MnP anode surface, Fig. 5e. The alloying strategy thus provides a clear route to suppress HER at the electronic structure level, with MnP delivering markedly prolonged cycling stability compared with pure Mn metal. Soundharrajan et al. proposed a manganese/iodine battery with AC (active carbon) ||Zn-Mn hybrid full-cell configuration. The authors utilized sodium iodide (NaI) as a redox-active additive in the Mn(ClO_4_)_2_ electrolyte. The full cell achieved an admirable electrochemical property. However, the primary working mechanism of Zn-Mn alloy relies on the deposition/dissolution of Zn, and the role of Mn during working needs further investigation [93]. In fact, Mn deposition is a complex process. Chae’s group provided insights on various factors, such as anion types, concentrations of the electrolyte, substrates, and cathode chemistries, that yielded different results in Mn metal batteries [94], however, further efforts are still needed to develop a more comprehensive understanding for achieving stable Mn deposition. These studies emphasize that stable Mn plating/stripping depends on multiple coupled factors, reinforcing the need for systematic investigations into electrolyte–electrode interactions to improve cycle life. Overall, Mn metal anodes face intrinsic challenges of low Coulombic efficiency and poor reversibility, primarily due to severe hydrogen evolution and uneven deposition. These limitations not only reduce cycle life but also hinder the practical feasibility of Mn anodes. Addressing these issues will require integrated strategies that combine electrolyte regulation, interfacial engineering, and alloying approaches to achieve stable and efficient Mn deposition.

### Intercalation-Type and Conversion-Type Anodes

Though recent studies have employed Mn metal as anode, low efficiency and poor cycling stability still exist in aqueous electrolytes. Thus, intercalation-type and conversion-type anodes as supplementary options are indispensable for AMIBs. In 2022, Nimkar et al. reported a Chevrel-based Mo_6_S_8_ as the anode for Mn^2+^ ion insertion [34]. In a saturated MnCl_2_ solution, Mo_6_S_8_ delivered a high capacity exceeding 90 mAh g^−1^ at 0.5 A g^−1^ and stable cycling performance with > 96% capacity retention over 1500 cycles at 5 A g^−1^. Importantly, proton insertion was observed in this Mo_6_S_8_ anode for the first time and contributed to enhanced storage capacity. Computational results depicted the migration pathway where Mn^2+^ ions diffused in the Chevrel structure via two competing pathways: (1) Circular motion within six equivalent tetrahedral sites of a cavity and (2) diffusion between adjacent cavities. The migration barriers across pathways demonstrated that Mn^2+^ ions would move in a circular motion in the inner ring unless higher polarization to more negative potentials was applied.

**Fig. 6:**
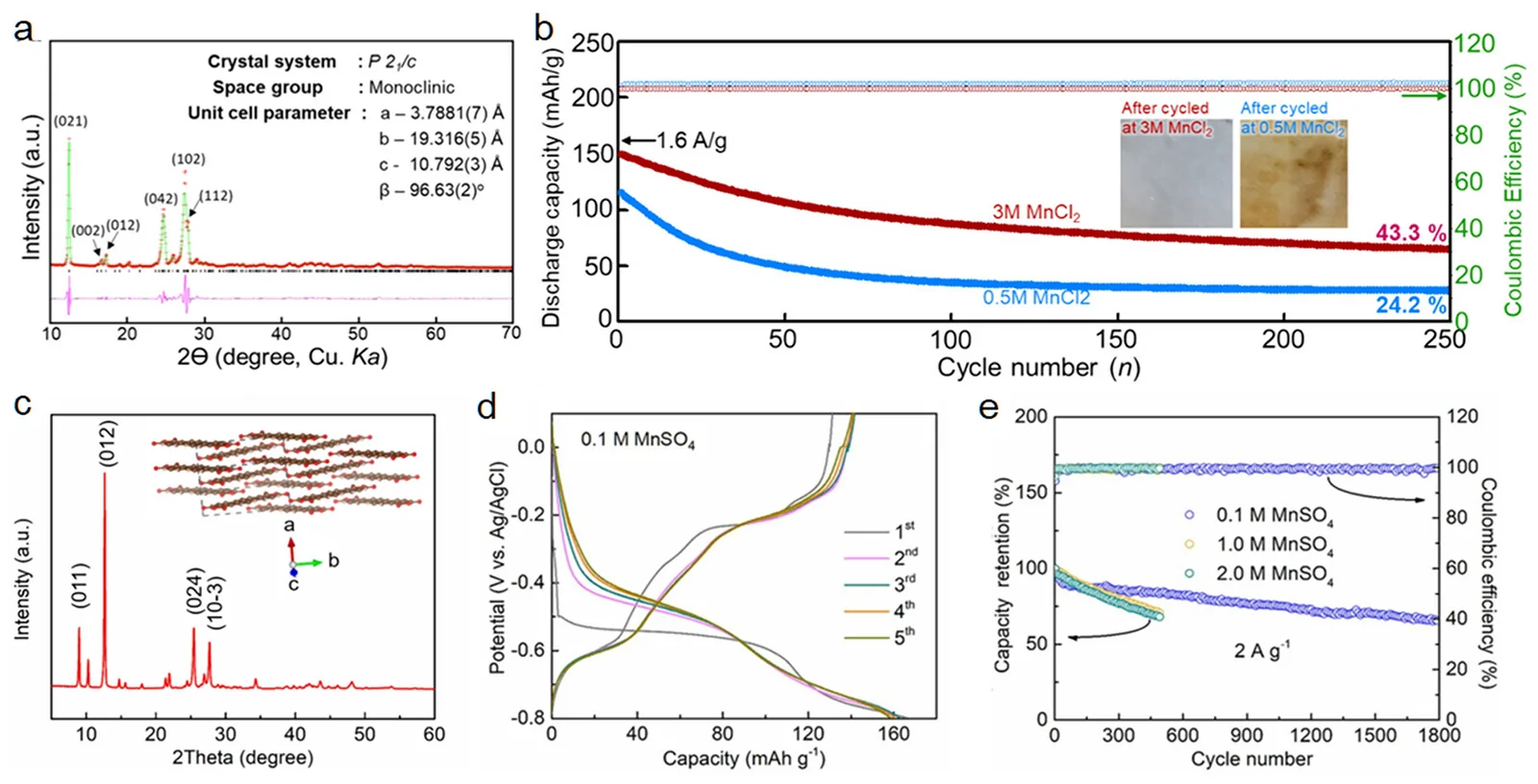
**a** Powder X-ray diffraction Rietveld refinement of *β*-PTCDA. **b** Cycling stability of *β*-PTCDA electrodes with separator images after cycling in different solutions. **a, b** Reproduced with permission [33]. **c** XRD pattern of *α*-PTCDA (inset is the diagram of crystal structure). **d** GCD curves in 0.1 M MnSO_4_, and **e** cycling stability at 2.0 A g^−1^ in 0.1, 1.0, and 2.0 M MnSO_4_ of *α*-PTCDA. **c–e** Reproduced with permission [60]

Recently, the organic compound perylene-3,4,9,10-tetracarboxylic dianhydride (PTCDA) was reported to accommodate Mn^2+^ ions as anode materials [33, 60]. As proposed by Lee et al., the carbonyl groups and the π-electron configuration endowed *β*-PTCDA (according to the characterizations offered in the paper, Fig. 6a) with reversible storage of Mn^2+^/H^+^, resulting in a capacity of 185 mAh g^−1^ at 0.1 A g^−1^ and enhanced cycling stability in a high-concentration electrolyte (3 M MnCl_2_, Fig. 6b) [33]. In another work, Dong et al. reported Mn^2+^ ions’ storage in *α*-PTCDA (Fig. 6c, d), and better cycling performance was achieved (76.9% capacity retention at 2.0 A g^−1^ after 1000 cycles) in low-concentration electrolyte, Fig. 6e [60]. Although both phases of PTCDA underwent enolization reaction during charge/discharge, the difference in electrochemical performance, probably arising from the structural difference, offers more insights into the underlying electrochemical mechanisms. These findings illustrate how organic anodes, despite being at an early stage, can deliver both reasonable capacities and prolonged cycling life with valuable insights into the role of molecular configuration.

**Fig. 7:**
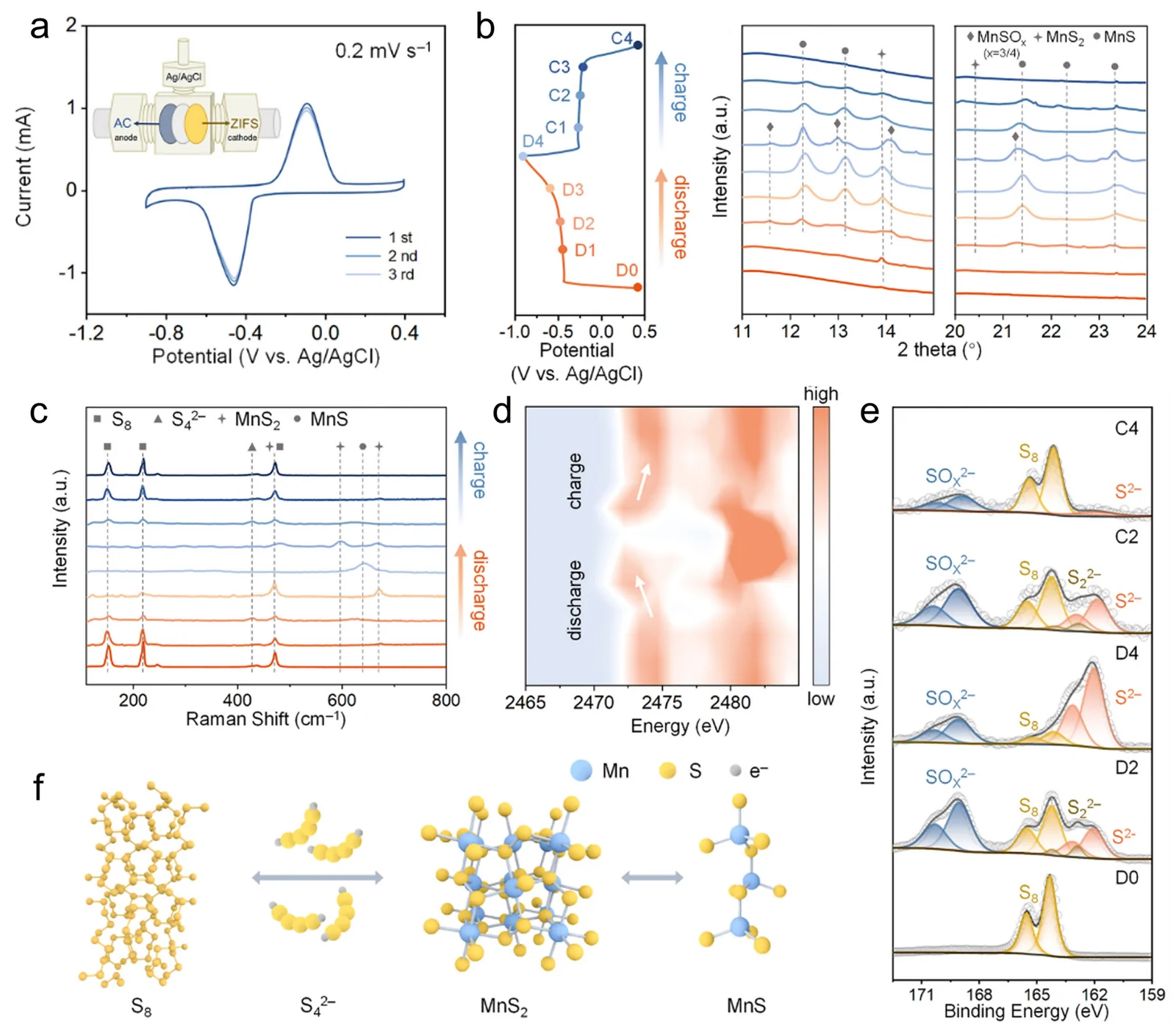
**a** CV curves of the half cell at 0.2 mV s^−1^. The inset illustrates the construction of the half cell. **b** Ex situ SXRD and **c** Raman spectra at selected states. **d** *Operando* S K-edge XAFS spectra in topographic view. **e** Ex situ XPS spectra of S 2*p*. **f** Schematic diagrams of the complete conversion pathways. **a–f** Reproduced with permission [58]

Sulfur (S) has been widely adopted as cathode material in other battery systems for its high theoretical capacity, non-toxicity, and natural abundance [95, 96]. However, many drawbacks still need to be resolved, including low electric conductivity and large volume expansion. More critically, the shuttling effect due to the solubility of metal polysulfides severely deteriorate the battery performance [95]. Tremendous efforts have been devoted to improve capacity values and cycling life, whereas there has been no report of utilizing S in AMIBs until very recently Chao’s group proposed the usage of S anode based on an energetic Mn^2+^-S redox electrochemistry [58]. The authors pioneered a solid–liquid–solid reaction pathway (S_8_ → soluble MnS_4_^2−^ → solid MnS_2_ → MnS), with its mechanism rigorously validated through *operando* X-ray absorption fine spectroscopy (XAFS) and synchrotron X-ray diffraction. In detail, as shown in Fig. 7a, CV results revealed distinct redox peaks at − 0.46 and − 0.2 V (vs. Ag/AgCl), such minimal voltage hysteresis (0.26 V) indicated favorable kinetics. Ex situ synchrotron XRD (SXRD) captured the disappearance of amorphous S_8_, followed by the emergence of MnS_2_ at mid-discharge state and *γ*-MnS at full discharge state, with complete reversibility upon charging, Fig. 7b. Further, ex situ Raman spectroscopy (Fig. 7c), *operando* S K-edge XAFS spectra (Fig. 7d), and ex situ XPS (Fig. 7e) identified the stepwise transformation with intermediates of S_4_^2−^ and MnS_2_ before *γ*-MnS dominated, the transformation cycle is displayed in Fig. 7f. Critically, the shuttling effect—a persistent issue in metal–sulfur systems—was mitigated by a polyvinyl alcohol (PVA) hydrogel membrane. The PVA’s hydroxyl groups chemically anchored polysulfides, while its uniform microstructure promoted homogeneous Mn^2+^ ions transport, evidenced by in situ electrochemical holography. This dual function suppressed S loss and enhanced reaction kinetics, enabling 88% capacity retention after 250 cycles at 2 A g^−1^. When coupled with a high-voltage MnO_2_/Mn^2+^ cathode (1.23 V vs. Ag/AgCl), the full cell achieved an energy density of 396 Wh kg^−1^ (based on the total mass of S and MnO_2_) and stable operation for 800 cycles at 4 A g^−1^. This study not only represents the first report of S anode in AMIBs, but also demonstrates how interfacial engineering with polymer membranes can effectively suppress shuttle effects and enable extended cycling stability, opening a new direction for novel anode materials.

In summary, anode development for AMIBs is still at an early stage, with research efforts spanning Mn metal, alloy, interfacial engineering, intercalation hosts, organic compounds, and even sulfur-based systems. Existing studies consistently indicate that anode performance is strongly governed by interfacial reactions, solvation–desolvation behavior of Mn^2+^, and the intrinsic instability of the Mn–water interface under low-potential conditions. For Mn metal anodes, severe hydrogen evolution, uneven deposition, and parasitic side reactions originate from the coupled effects of surface chemistry, and electrolyte composition. Notably, the industrial electrolytic manganese metal process has long confronted similar competition from hydrogen evolution and deposit-quality instability. To this, diverse strategies have been put forward, including electrolyte buffering/acidity control and process-condition optimization (e.g., high salt and low H^+^ environments with controlled current density and mass transport) [97, 98]. These industrially validated principles may provide guidance for mitigating HER and improving Mn plating/stripping reversibility in AMIBs. Although notable progress has been achieved through alloying, protective interphases, and electrolyte regulation, challenges related to Coulombic efficiency and long-term reversibility remain unresolved. For non-metal anodes, these materials can partially bypass direct Mn plating/stripping and thus alleviate the most aggressive low-potential instability, enabling comparatively stable cycling in several reported systems. However, the capacity of these materials is relatively lower, and their reaction chemistries remain diverse and case-dependent, leading to limited systematic understanding. Compared with cathodes, anode research is clearly less mature, yet it represents a decisive bottleneck for the overall performance of AMIBs. Future anode development should therefore emphasize electrolyte–anode co-design and systematic mechanistic investigations to clarify deposition dynamics and interfacial evolution, thereby enabling stable and practical long-term cycling in AMIBs.

## Electrolyte for AMIBs

The electrolyte in an aqueous battery serves as the fundamental medium enabling the core electrochemical processes, acting as a critical determinant of overall cell stability and performance. It bears the responsibility to transmit charge carriers between cathode and anode during charge and discharge cycles. Beyond simple ionic conduction, electrolytes dictate the electrochemical stability window (ESW), govern reaction kinetics at both electrodes, influence interfacial stability, and critically impact parasitic side reactions that degrade cell performance [99–101]. Thus, electrolyte engineering is beneficial for enhancing the electrochemical performance of AMIBs. So far, several studies have arisen for both intercalation—electrodes and Mn metal anodes. Key strategies include optimizing Mn salt concentration and selecting appropriate supporting salts, carefully controlling pH to balance Mn deposition/dissolution against HER, and employing functional additives or co-solvents to alter solvation structures and mitigate HER. These different electrolyte design approaches collectively demonstrate that tuning solvation structures, ion coordination, and interfacial chemistry is as important as electrode optimization for achieving stable AMIB performance.

### Electrolyte Modulation for Mn Metal Anode

**Fig. 8:**
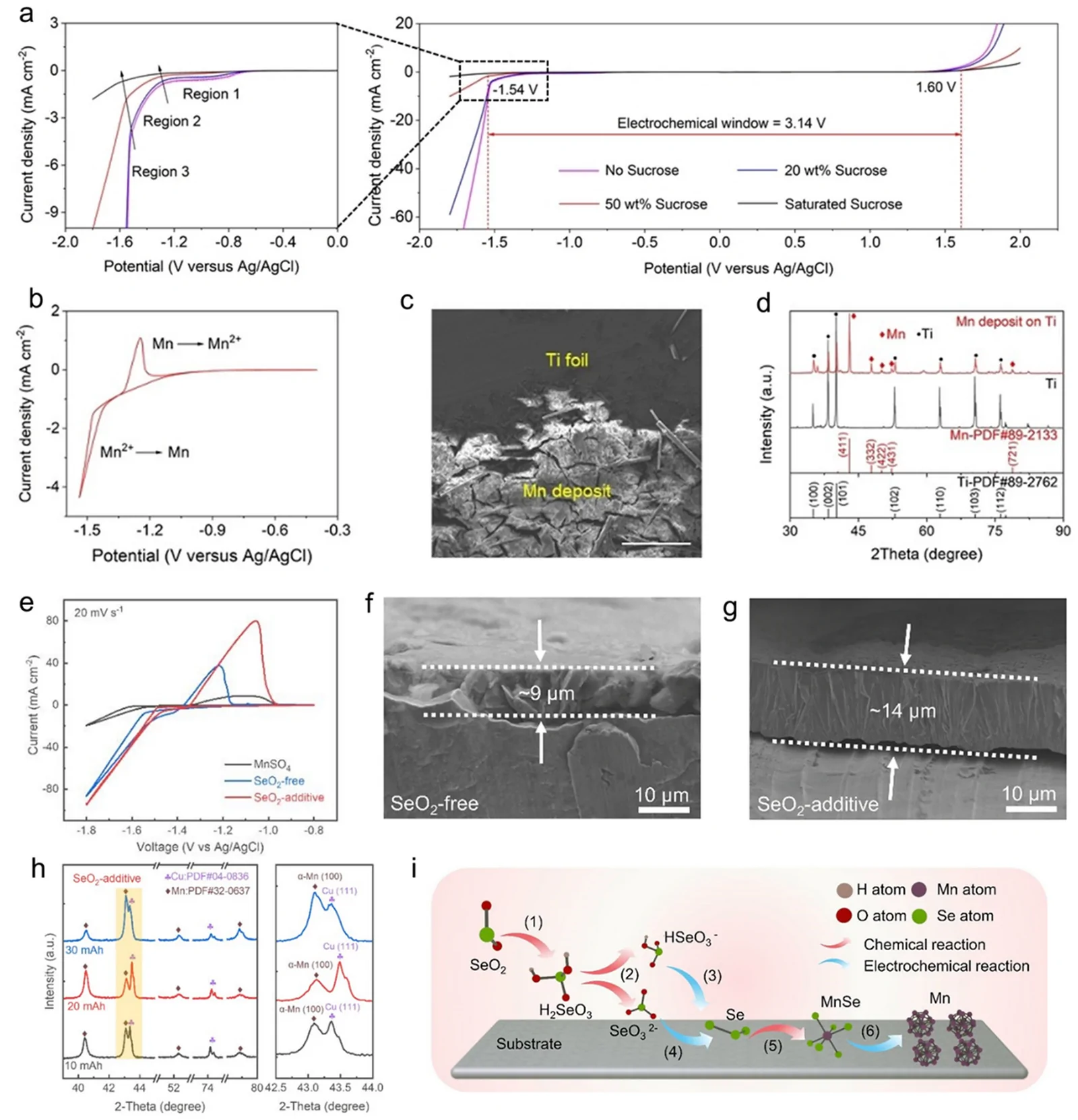
Physicochemical property characterization of aqueous electrolytes. **a** ESW of aqueous electrolytes with sucrose, NaClO_4_, and glycine examined by LSV at 10 mV s^−1^. The anodic region is magnified to show the detailed reactions. **b** CV curve tested in a three-electrode system using Ti as working electrode in the 50 wt% sucrose electrolytes. **c** SEM image and **d** XRD data of Ti electrode after plating Mn. **a–d** Reproduced with permission [43]. **e** CV comparison in different electrolytes. Cross-section SEM of deposited Mn in **f** SeO_2_-free and **g** SeO_2_-additive MnSO_4_ electrolyte. **h** XRD of deposited Mn on Cu foil in SeO_2_-additive electrolyte. **i** Schematic diagram of the Mn deposition process in the SeO_2_-additive electrolyte. **e–i** Reproduced with permission [37]

Electrolyte modulation is of high significance for Mn metal anode as HER will be highly pronounced and competitive over metal deposition reaction at low potentials. Under this condition, limiting the reactivity of water and promoting Mn deposition/dissolution are the primary objectives when a metal anode is applied. Zhi’s group proposed a MnSO_4_–NaClO_4_–glycine–sucrose mixed solution to decrease the activity of water and improve anodic stability at low potentials [43]. In detail, sucrose with 50 wt% addition prominently restrained the activity of water molecules, effectively expanding the electrochemical stability window (Fig. 8a**)**. FTIR spectra showed the shift of O–H bond, indicating that sucrose molecules formed extensive hydrogen-bond networks with H_2_O, lowering its chemical activity and thereby suppressing HER. The CV profiles (Fig. 8b) exhibited a pair of typical plating and stripping peaks. Scanning electron microscope (SEM) (Fig. 8c) and XRD (Fig. 8d) further proved the deposition of Mn. Nevertheless, it was observed that the area of cathodic peak was larger than anodic peak, indicating the side reaction of water decomposition during Mn plating, which was inevitable even though the electrolyte was carefully designed. Consequently, a 250-h long cycling lifespan of Mn symmetric cell and 200 cycles without capacity decay were achieved, indicating its advances for Mn metal anode.

In 2022, Su’s group reported a MnO_2_||(0.1 M MnSO_4_ + 0.5 M H_2_SO_4_)|(0.00025 M H_2_SO_4_ + 0.1 M MnSO_4_)||Mn all-manganese battery via an anion-exchange membrane to achieve acidity adjustment [41]. Through adjusting the pH of anolyte to a fairly low H_2_SO_4_ concentration, HER turned to be ignorable. The same method was applied to the catholyte so that the redox potential of MnO_2_/Mn^2+^ reached 1.071 V versus standard calomel electrode (SCE). Owing to the high electrode potential of MnO_2_ and low potential of Mn metal, the open circuit voltage and operating voltage reached 2.383 and 2.1 V, respectively.

Electrolyte-induced interfacial engineering is another strategy for suppressing HER and promoting uniform deposition. Beside using sucrose to reduce the reactivity of water, Zhi’s group demonstrated that the MnSO_4_–NaClO_4_–glycine–sucrose mixed solution helped to construct an organic interface via esterification reaction [43] (details have been discussed in the **Mn Metal Anode** section). This organic layer at the electrolyte–electrode interface not only enabled a bubble-free interface as a dense shield but permitted Mn diffusion. Consequently, a 250-h long cycling lifespan of Mn symmetric cell and 200 cycles without capacity decay were achieved. Indicating its advances for Mn metal anode. Such results clearly indicate that electrolyte-induced interfacial layers can be an effective engineering strategy to stabilize Mn plating/stripping, extending cycling stability compared with unmodified systems.

In another work, Chen and collaborators developed an aqueous all-Mn battery [37]. Inspired by industrial metallic Mn electrodeposition, a small amount of SeO_2_ additive was introduced into a MnSO_4_/(NH_4_)_2_SO_4_ mixed solution. The added (NH_4_)_2_SO_4_ acted to buffer the pH and alternated the coordination environment of Mn^2+^ ions, and SeO_2_ additive reduced the overpotential of Mn plating/stripping and improved the Coulombic efficiency (indicated by the CV profiles in Fig. 8e**)**. The authors revealed that during the nucleation stage, the SeO_2_ additive promoted the generation of Se and MnSe intermediates, which acted as preferential nucleation centers for metallic Mn. This reduced the deposition overpotential and guided uniform deposition, enabling a more homogeneous and denser deposition of Mn (as shown in SEM images, Fig. 8g) and preventing severe deposited Mn from oxidation. Thus, a thicker layer of Mn was obtained, compared with that in SeO_2_-free electrolyte, Fig. 8f. XRD pattern (Fig. 8h) indicated that the deposited Mn adopted *α*-phase with a more stable crystal structure conducive to the anode reversibility. The working mechanism of this additive was proposed (Fig. 8i), and the overall electrochemical reaction was detected and elucidated below. Such results clearly indicate that both electrolyte-induced interface engineering is an effective strategy to stabilize Mn plating/stripping.

4 $$\mathrm{SeO}_{2} +\mathrm{H}_{2}\mathrm{O}\leftrightarrow \mathrm{H}_{2}\mathrm{SeO}_{3}$$

5 $$\mathrm{H}_{2}\mathrm{SeO}_{3}\leftrightarrow \mathrm{HSeO}_{3}^{-}+\mathrm{H}^{+}\leftrightarrow Se{O}_{3}^{2-}+{2H}^{+}$$

6 $$\mathrm{HSeO}_{3}^{-}+2\mathrm{H}_{2}\mathrm{O}+4{e}^{-}\to \mathrm{Se}+5\mathrm{OH}^{-}$$

7 $$\mathrm{SeO}_{3}^{2-}+3\mathrm{H}_{2}\mathrm{O}+4{e}^{-}\to \mathrm{Se}+6\mathrm{OH}^{-} $$

8 $$\mathrm{Se}+2{e}^{-}\to \mathrm{Se}^{2-}$$

9 $$\text{ Se}^{2-}+\mathrm{Mn}^{2+}\to \mathrm{MnSe} $$

While aqueous electrolytes will inevitably induce HER during low-potential deposition, organic electrolytes provide a parallel strategy through coordination structure regulation. Chen’s group reported a halogen-mediated nonaqueous electrolyte to reduce plating/stripping overpotentials of Mn metal anode [102]. Chlorine atoms with lower electronegativity were introduced into the Mn^2+^ solvated clusters, resulting in a weaker interaction between Mn^2+^ ions that improved the coulombic efficiency. Shen et al. proposed a novel design of a Mn-based dual-storage mechanism in the same organic electrolyte [36], and the Mn||Cu_1.8_S full cell cycled stably over 400 times at 500 mA g^−1^ with 83.3% capacity retention. Beyond this halogen-mediated electrolyte, Jing et al. elucidated the fundamental link between Mn^2+^ solvation and interfacial behavior in a nonaqueous electrolyte with 2-methoxyethylamine (MOEA) additive [103]. The authors transformed the solvation shell into Mn–N-dominated complexes that weaken Mn–solvent interactions and lower desolvation barriers. This polarizable coordination environment accelerated Mn^2+^ reduction kinetics and stabilized deposition, enabling stable cycling over 2000 h. These works highlight how molecular-level solvation tuning can achieve highly reversible Mn metal anodes, providing valuable suggestions for AMIBs.

Alongside liquid electrolytes, gel polymer electrolytes provide an alternative pathway to mitigate water-induced parasitic reactions. Du’s group designed a Mn^2+^ cross-linked sodium alginate gel electrolyte (SA@Mn) [62]. The hydrophilic polymer chains reduce the free water content, inhibiting water-related parasitic reactions and corrosion. Meanwhile, the gel electrolyte leveraged ionic cross-linking interactions between alginate chains and Mn^2+^, which builds Mn^2+^ migration pathways. The optimized SA@Mn gel exhibited a high ionic conductivity of 172.5 mS cm^−1^ and a Mn^2+^ transference number of 0.89 at ambient temperature. As a result, the Mn||Mn symmetric cell achieved stable plating/stripping for > 450 h, and a quasi-solid-state Mn metal pouch cell was successfully fabricated, highlighting the feasibility of gel-based designs for practical Mn metal batteries.

### Electrolyte Modulation for Other Electrode Materials

The significance of electrolyte is reflected not only in the usage of Mn metal anode but also in the impact on intercalation/conversion-type electrode materials. In aqueous environment, the size of metal ions is determined by their hydrated radius rather than ionic radius, and ion–water interactions can be described as M^z+^(H_2_O)_n_, where the hydration shell is geometrically defined by the number of water molecules [104]. As in conventional aqueous solutions, Mn^2+^ ions exist as Mn^2+^(H_2_O)_6_ [105] and the corresponding hydrated radius is larger than that of Zn^2+^ and Mg^2+^ [32], which poses challenges to electrode materials. Thus, it is a valid method of regulating the solvation environment to improve electrode performance when adopting intercalation-type electrodes. For example, highly concentrated “water-in-salt” electrolytes (WISEs) have shown their unique advantages in aqueous systems [106], and their potential application was first demonstrated by the use of saturated MnCl_2_ [34]. As proposed, a more acidic environment (pH = 1.4 for saturated vs. pH = 4 for 1 M MnCl_2_) enabled proton insertion into Chevrel-phase Mo_6_S_8_, resulting in an enhanced capacity even though the conductivity of the saturated electrolyte was relatively lower.

As mentioned in the previous section, anion types will exert influence on cell performance, and this phenomenon is also widely observed in ZIBs [12, 107, 108]. Similarly, different choices of manganese salts significantly affect the actual behaviors of Mn batteries. Niu’s group investigated the differences of MVO cathode in MnSO_4_ and Mn(CF_3_SO_3_)_2_ aqueous electrolytes and observed a fast capacity degradation in MnSO_4_ [32]. Although the electrochemical windows of MnSO_4_ and Mn(CF_3_SO_3_)_2_ were similar, Mn(CF_3_SO_3_)_2_ electrolyte demonstrated improved wettability toward the cathode, which facilitated better electrolyte infiltration into the active materials. Meanwhile, bulky CF_3_SO_3_^−^ anions in the electrolyte reduce the coordination number of water and solvation effect, facilitating ionic mobility of charge carriers. This comparison emphasizes how specific anions influence electrode wetting and cycling stability, underlining the necessity of carefully matching electrode materials with suitable salts.

**Fig. 9:**
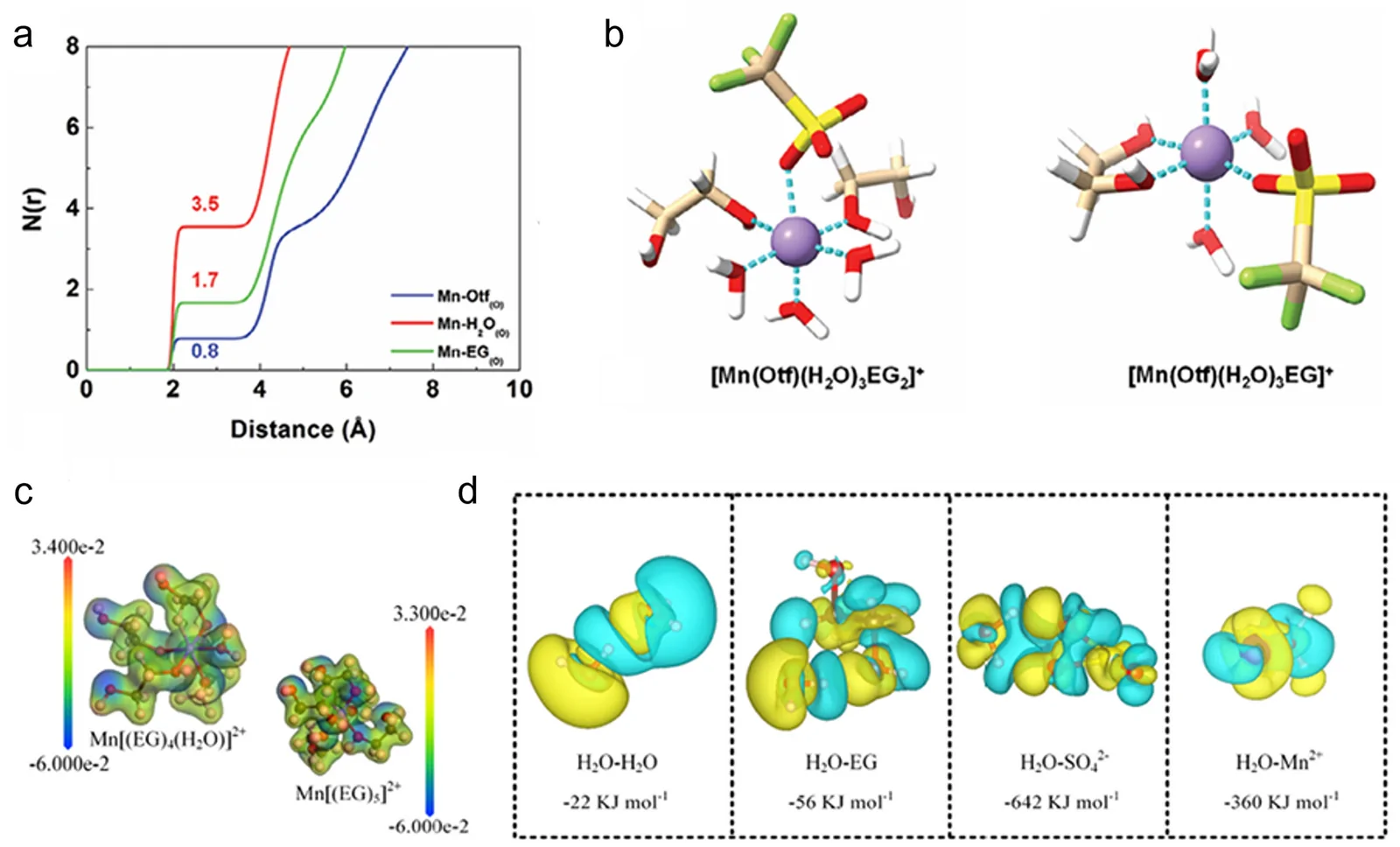
**a** Coordination number N(r) of Mn–O (Otf, H_2_O, EG) in 50% EG electrolyte **b** Solvated structure of Mn^2+^ ion in 50% EG electrolyte. **a, b** Reproduced with permission [51]. **c** Molecular electrostatic potential energy surface of [Mn(EG)_4_(H_2_O)]^2+^ and [Mn(EG)_5_]^2+^. **d** Difference charge density and calculated binding energies of H_2_O-H_2_O, H_2_O-EG, H_2_O-SO_4_^2−^, and H_2_O-Mn^2+^. The EG is easily combined with H_2_O compared with the combination between H_2_O, Mn^2+^, and SO_4_^2−^. The yellow and blue regions represent the accumulation and depletion of electrons, respectively, suggesting the effective interactions between them. **c, d** Reproduced with permission [111]

Introducing co-solvents or other components is another strategy to tune the solvation cluster [109, 110]. Mai’s group introduced polar ethylene glycol (EG) as a co-solvent to achieve more reversible Mn^2+^/proton storage [51]. One EG molecule has two pairs of hydrogen-bond (HB) donors and acceptors, which can participate in EG-water HB formation with lower binding energy. Compared to pure 1 M Mn(CF_3_SO_3_)_2_ aqueous electrolyte where Mn^2+^ mainly existed in the [Mn(H_2_O)_6_]^2+^ and [Mn(Otf)(H_2_O)_5_]^+^ forms, solvated Mn^2+^ ions were mainly present in the [Mn(Otf)(H_2_O)_3_EG_2_]^+^ and [Mn(Otf)(H_2_O)_3_EG]^+^ forms in 50% EG hybrid electrolyte according to the radial distribution function (RDF) results (Fig. 9a, b). In addition, MD simulations indicated a sharp decrease in H_2_O − H_2_O HB proportion in 50% EG electrolyte compared with that in aqueous electrolyte, indicating a reconstruction of HB network that reduced the rapid proton transfer and prevented uncontrolled Grotthuss proton conduction. Moreover, the addition of EG also modulated the electric-double-layer state of the VO_2_ electrode interface, preventing protons and H_2_O molecules from accumulating on the surface, thereby inhibiting the vanadium dissolution. Likewise, Shi et al. employed EG as a molecular crowding agent to alter hydrogen-bond structure (Fig. 9c, d) at a lower salt concentration [111]. Based on such electrolyte design, aqueous Mn-ion hybrid microsupercapacitor (hydroxylated Ti_3_C_2_T_x_ MXene anode and Al_*x*_V_2_O_5_ with dimethylformamide-intercalated cathode) exhibited high-energy density (138.64 µWh cm^−2^), power density (10.0 mW cm^−2^) and long cycle life (99.7% after 5000 GCD cycles).

**Fig. 10:**
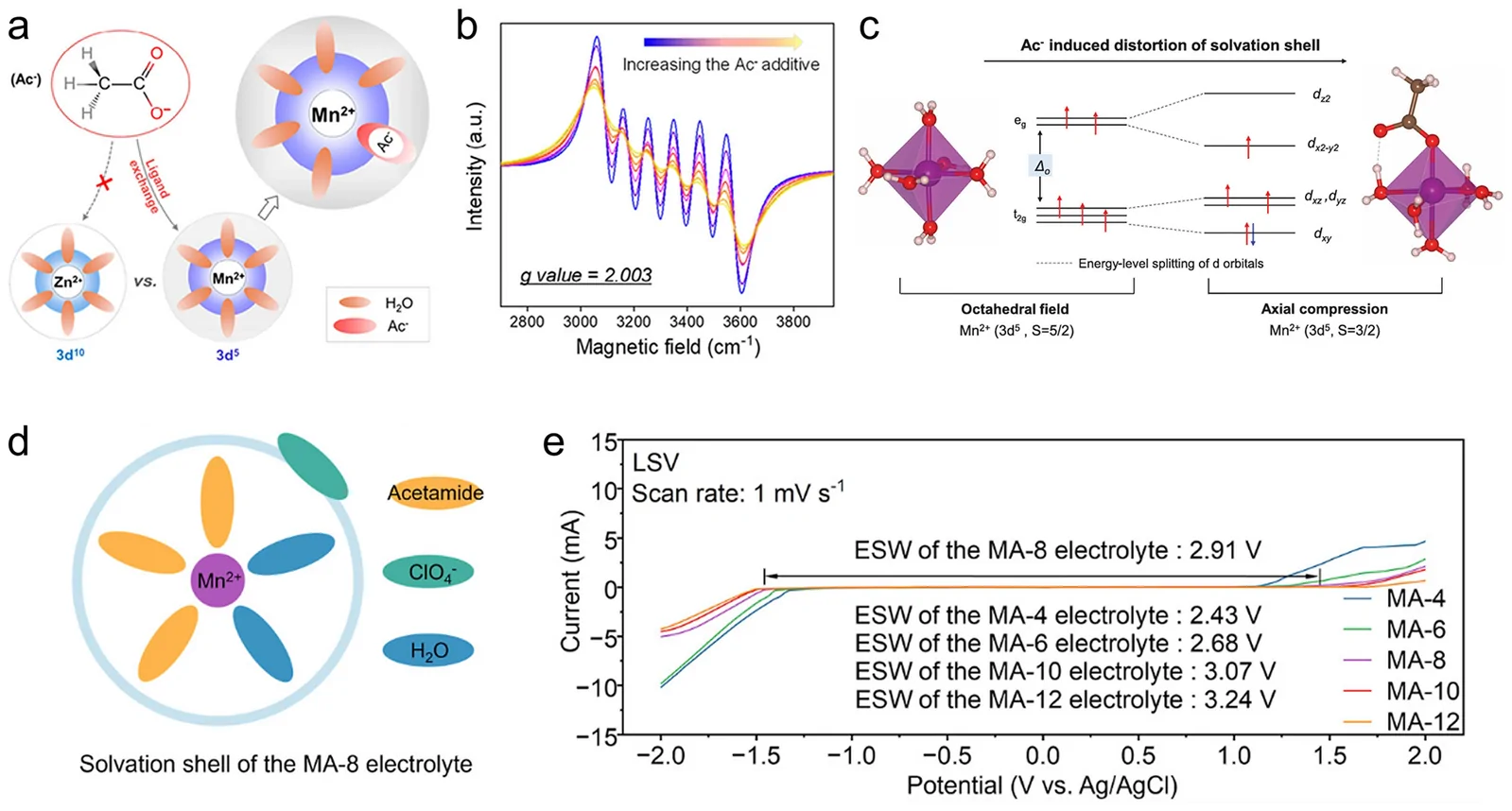
**a** Illustration of the effect of low-concentration Ac^−^ on the solvation sheath of Zn^2+^ and Mn^2+^ in an aqueous solution. **b** ESR spectra of MnCl_2_ solutions at varying Ac^−^ concentration (concentrations of [MnCl_2_:MnAc_2_] are [1:0], [0.9:0.1], [0.75:0.25], [0.5:0.5], [0.25:0.75], [0.1:0.9], and [0:1], scale bar from purple to yellow). Upon the addition of the Ac^−^ ligand, the representative signal of [Mn(H_2_O)_6_]^2+^ begins to diminish, suggesting that Ac^−^ penetrates the coordinating solvent layer of Mn^2+^ and induces d-electron rearrangement of the Mn^2+^. **c** *d*-orbital energy level splitting and electron rearrangement of Mn^2+^ induced by Ac^−^ coordination. When the Ac^−^ ligand is introduced, it bonds to Mn^2+^ with a shorter bond length, which creates significant repulsion with the d*z*^2^ electron, thus raising the dz^2^ energy level while lowering the d_*x*_^2^_–*y*_^2^ energy level, ultimately removing the degeneracy of the e_g_ orbitals. **a–c** Reproduced with permission [63]. **d** Illustration of solvation shell of the eutectic electrolyte. **e** LSV curves of electrolytes with different compositions. **d, e** Reproduced with permission [64]

Cai et al. demonstrated an ion-specific coordination strategy utilizing acetate anions (Ac^−^) to reconstruct the solvation structure of Mn^2+^ ions (Fig. 10a) [63]. Unlike conventional metal ions (e.g., Zn^2+^), the half-filled 3*d*^5^ electron configuration of Mn^2+^ enables unique sensitivity to low-concentration Ac^−^. Electron paramagnetic resonance (EPR) spectrum of the 1.0 M MnCl_2_ electrolyte, displayed in Fig. 10b, exhibited distinct peaks centered at g = 2.003, corresponding to a high-spin octahedral crystal-field configuration of [Mn(H_2_O)_6_]^2+^. With the introduction of Ac^−^, the intensity of the representative peaks gradually decreased as the concentration of Ac^−^ increased, suggesting d-orbital splitting and electron rearrangement induced by Ac^−^ (Fig. 10c). Raman spectroscopy demonstrated the destruction of octahedral hydrated coordination configuration by Ac^−^, and confirmed the reorganization of hydrogen-bond networks. Both results explained the penetration of Ac^−^ into the primary solvation shell. RDFs results showed an elongated average Mn–O(w) bond and reduced water coordination number, while DFT calculations further confirmed the spontaneous entry of AC^−^ into the inner hydration shell of Mn^2+^, replacing one coordinating water molecule with thermodynamically favorability. Consequently, the desolvation kinetics were significantly enhanced by this coordination shift. DFT analysis showed the energy barrier for removing the first H_2_O molecule dropped from + 0.07 eV in [Mn(H_2_O)_6_]^2+^ to − 0.003 eV in [MnAc(H_2_O)_5_]^+^. Galvanostatic intermittent titration technique (GITT) revealed a two-order-of-magnitude increase in Mn^2+^ diffusion coefficient (10^–12^ → 10^–10^ cm^2^ s^−1^) during deep intercalation (> 50% state of charge) in perylenetetracarboxylic diimide (PTCDI) anodes. This ion-specific solvation modulation enabled a two-order-of-magnitude increase in Mn^2+^ diffusion coefficient and excellent cycling retention of 96.3% after 1000 cycles at 1.0 A g^−1^ in full cells, underscoring the effectiveness of targeted solvation control strategies.

Besides, Jin’s group first presented a series of hydrated eutectic electrolytes composed of Mn(ClO_4_)_2_·6H_2_O and acetamide (denoted as MA-x, where x refers to the molar ratio of Mn^2+^ to acetamide). MD simulations confirmed that acetamide progressively replaced H_2_O molecules in the Mn^2+^ solvation shell (illustrated in Fig. 10d**)** [64]. The electrolyte suppressed water reactivity via reconstructed hydrogen-bonding networks, achieving a widened electrochemical window (2.91 V) in Fig. 10e. Based on this carefully designed electrolyte, the assembled PTCDI||NiHCF full cell achieved enhanced cycling stability with 95.6% retention after 1200 cycles, exemplifying how unconventional electrolyte chemistries can significantly enhance stability.

Besides, electrolyte modulation enables a unique redox chemistry based on the Mn^3+^/Mn^2+^ couple. Li et al. reported a regulated electrolyte of 3 M H_2_SO_4_/0.5 M MnSO_4_/0.1 M SnSO_4_/0.05 M Na_4_P_2_O_7_ that enabled high-potential Mn^3+^/Mn^2+^redox reaction [44]. Pyrophosphate ligand aided in building a protective layer via a complexation reaction on the electrode surface, which effectively prevented Mn^3+^ from undergoing disproportionation and restrained the uncontrollable diffusion of Mn^3+^ into the electrolytes. As a result, the Sn||AGrE (activated graphite electrode) full battery exhibited remarkable electrochemical performance, including a large capacity of 0.45 mAh cm^−2^ at 5 mA cm^−2^, excellent rate capability and superior cycling stability with no capacity decay over 30,000 cycles. Very recently, Yuan et al. introduced a synergistic strategy involving coordination optimization with ethylenediamine tetraacetate anions (EDTA^4−^) to stabilize Mn^3+^ and interfacial confinement to reduce Mn^3+^ migration [45]. The results showed that this approach significantly improved the Mn^3+^/Mn^2+^ redox reversibility, achieving an areal capacity of 0.36 mAh cm^−2^ at 4 mA cm^−2^ with 81% capacity retention over 3000 cycles.

In summary, electrolyte engineering represents a primary lever for regulating the intrinsic physicochemical constraints of manganese redox chemistry in aqueous systems. Through solvation shell reconstruction, coordination modulation, and interfacial chemistry control, electrolyte formulations can effectively tune the desolvation kinetics of Mn^2+^, suppress water-induced parasitic reactions and influence the stability of intermediate Mn^3+^ species. Strategies including high-concentration electrolytes, pH regulation, functional additives, co-solvent design, and eutectic systems collectively demonstrate how tailored chemical environments reshape reaction pathways at both electrodes. Nevertheless, these strategies also exhibit distinct trade-offs. High-concentration and eutectic electrolytes often suffer from increased viscosity and compromised ionic conductivity, whereas additive/co-solvent and pH regulation approaches provide more targeted control but may introduce composition complexity and formulation dependence. Such contrasts explain why electrolyte improvements are frequently chemistry specific. Thus, achieving universal long-term stability across different electrode chemistries remains challenging. Future advances will therefore rely on systematic electrolyte–electrode co-design and mechanistic understanding to balance electrochemical stability windows, ion transport, and interfacial robustness in practical AMIBs [24, 112].

## Conclusion and Perspectives

Aqueous manganese-ion batteries have shown their huge potential in energy storage fields due to low cost, high safety, and facile manufacturing processes. However, critical challenges require further efforts especially in the development of electrode materials and electrolyte optimization [56, 58, 63]. Indeed, failure of AIMs is largely system-level consequences of aqueous constraints. The water stability window imposes an inherent selectivity limit; low-potential processes inevitably compete with hydrogen evolution. This is explicitly manifested in Mn metal configurations, where severe HER and bubble formation directly degrade Coulombic efficiency and cycling stability. It explains why performance gains can be fragile when electrolyte/interface chemistry is not simultaneously stabilized. Besides, local chemical-environment change, especially pH gradients, can continuously reshape the true reaction interface and trigger secondary phase formation. It is known that manganese could react with water at low potential, leading to the generation of Mn(OH)_2_ passivation layer on their surface [24]. Such insulating layer could result in large polarization and limited cycling life. It is worth noting that this process is not anode specific, as its existence is also proven on the cathode side [35, 52]. Briefly, pH/OH^−^-driven reaction may alter kinetics and compromise reversibility. In this case, controls of interface and electrolyte should be emphasized, not merely single one of them.

Another critical limit falls on the chemistry of manganese. Mn^2+^ solvation and desolvation kinetics constitute a primary bottleneck. Strong hydration increases interfacial barriers that makes insertion disfavored. Strong static interaction of inserted Mn^2+^ could induce chemo-mechanical strain, leading to phase evolution and structural deterioration. Even when stress is not directly quantified, widespread use of in situ and ex situ structural tracking reflects continued attempts to verify insertion-driven structural impacts. Finally, although direct evidence of Mn^3+^-induced Jahn–Teller distortion is limited in many reported cathodes, its intrinsic electronic instability warrants explicit attention as a high-risk hypothesis guiding mechanistic validation and stability-focused design.

To accelerate the translation of AMIBs from laboratory demonstrations to practical application, it is crucial to elevate performance on device-level. At present, only a limited number of reports provide device-level projections, and the energy density obtained is not yet satisfactory. Niu’s group calculated the energy density of Mn||MVO full cell, which reached 64.8 Wh kg^−1^ energy density at 12.1 kW kg^−1^ (the mass of device is regarded as five times of the active materials in cathodes) [32]. Chen’ group reported that Mn||MnO_2_ full cell could reach 900 Wh kg^−1^ [37]. Mai’s group fabricated an all-vanadium pouch with mass loadings in the cathode and anode are ~ 5 and 15 mg cm^−2^ [51]. Du’s group fabricated a Mn||V_2_O_5_ full cell, which cycled over 200 cycles with a mass loading of 4 mg cm^−2^ [42]. However, these insufficient full cell data and mass-balancing information often prevents consistent comparison across studies. Moreover, electrochemical tests are still predominantly evaluated under low areal loadings, whereas practically relevant operation requires high areal capacities, limited electrolyte volumes, and robustness against temperature fluctuations. It is necessary to address this evaluation gap for identifying truly scalable chemistries and for guiding rational battery designs toward real-world conditions.

**Fig. 11:**
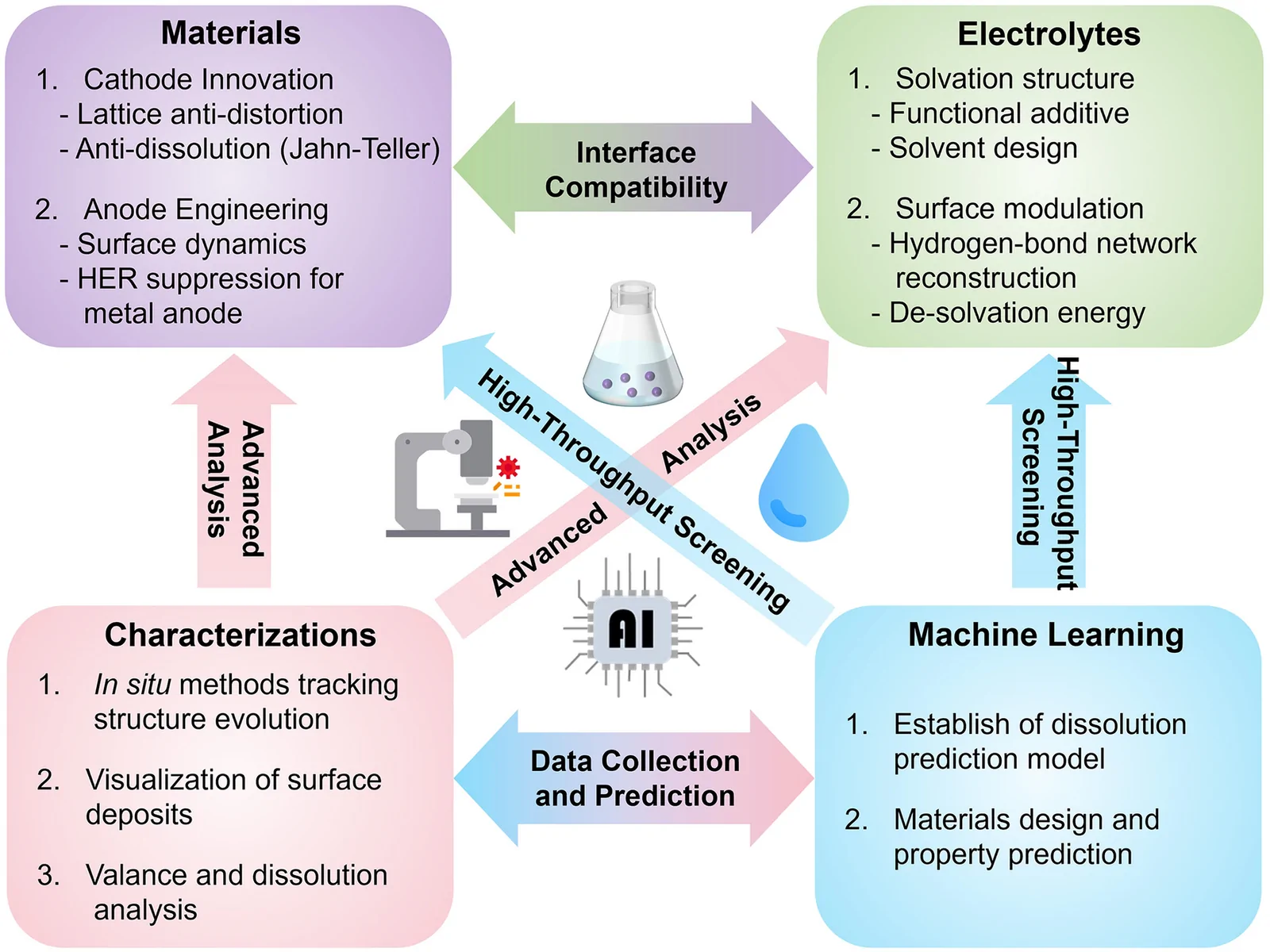
Future perspectives of advanced AMIB

Against this backdrop, we summarize key directions for the next stage of AMIB research (Fig. 11). These opportunities span electrode engineering, electrolyte design, mechanistic understanding, advanced characterization, and AI-driven discovery.

1. Exploring Novel Electrode Materials. Current cathode materials are predominantly vanadium-based oxides, while the choices for anode are much more limited. For intercalation-type electrodes, it should prioritize stable frameworks, sufficient redox-active sites, and moderated host–ion interactions. Among various materials, PBAs with large open frameworks show great potential for accommodating large ions. Notably, a high capacity can be achieved with tunable redox centers, and performance could be further improved through targeted structural optimization. Organic electrodes provide structural tunability and abundant chemistry, yet require solutions for conductivity and stability. Through co-insertion of protons, high capacity and energy density can be obtained. Metal organic frameworks (MOFs) and covalent organic frameworks (COFs) are promising due to high porosity and tunable metal sites, indicating rapid diffusion kinetics and low ion-induced strain. But their viability in AMIBs needs further systematic verification. Looking forward, electrode design must explicitly address the intrinsic challenges of Mn^2+^, particularly its large hydrated radius and strong electrostatic field. Future progress will rely on structure-tolerant frameworks, tunable organic hosts, and pre-intercalated stabilizers to buffer these effects, providing both high capacity and structural reversibility. Importantly, these electrode strategies should be validated under device-relevant areal loadings with balanced electrode mass, so that gains in gravimetric metrics can translate into practical energy density.
2. Regulating Electrolyte Composition. Electrolytes govern Mn^2+^ solvation, interfacial reactions, and transport. Multiple electrolyte modulation strategies have been put forward in other ABs, and these strategies are expected to be instructive and applicable for AMIBs. For intercalation-type electrodes, proper electrolyte modifications can effectively suppress the dissolution issue, alter the solvation shell, and adjust the Mn^2+^/proton insertion process. For Mn metal anodes, electrolyte optimization is indispensable to suppress HER, corrosion, dendrite growth and enable reversible plating/stripping, often via additives/co-solvents and interface-forming chemistries. Going forward, electrolyte research should move beyond single-variable optimization to integrated design principles that balance stability, ionic transport, and interfacial chemistry. Water-in-salt electrolytes, eutectic systems, functional additives, and co-solvent engineering each offer partial solutions, but systematic comparisons and hybrid strategies are needed to suppress HER, mitigate Mn dissolution, and regulate solvation simultaneously. Coupling electrolyte design with tailored electrode and interface modifications will be crucial, especially under limited electrolyte volume and temperature-relevant conditions, where gas evolution and long-term stability become more stringent.
3. Understanding the Underlying Mechanisms. Limited research generally means incomplete mechanistic insights into fundamental mechanisms. The complexity of Mn^2+^/H^+^ co-insertion, solvation shell dynamics, and side reactions makes it difficult to generalize mechanistic insights across different systems. Current studies often remain case-specific, and lack a universal framework that connects material chemistry, electrolyte environment, and electrochemical response. Although components in AMIBs may follow some shared principles, the selection of electrode/electrolyte appears to be limited and arbitrary without systematic guidance. Additionally, different materials and electrolyte compositions face diverse challenges that hinder full screening of the mechanisms. Systematic and standardized experiment and assessment processes are indispensable for comprehensive mechanisms. Theoretical studies have also provided indispensable support for understanding and optimizing AMIBs. DFT calculations have clarified electronic structures, adsorption energies, and reaction energetics at electrodes. MD simulations have revealed solvation environments, ion transport pathways, and hydrogen-bond network reconstructions. Finite element methods and related models have offered insights into stress distribution and interfacial stability. By complementing experimental observations, these theoretical approaches deepen the mechanistic understanding of Mn-ion storage and guide rational electrode and electrolyte design. Looking forward, the integration of multiscale simulations with advanced characterization and data-driven methods is expected to accelerate the discovery of new materials and establish design principles for high-performance and durable AMIBs.
4. Utilizing Advanced Characterizations. In situ and multimodal characterization is essential to capture dynamic phase evolution, interfacial chemistry, and reaction intermediates. Advanced characterizations (inelastic neutron scattering, nuclear magnetic resonance (NMR) spectroscopy, X-ray absorption spectroscopy (XAS), etc*.*) and in situ characterizations (FTIR, Raman, XRD, etc*.*), can establish critical connections between electrochemical performance and microstructural/chemical evolution of electrodes and electrolytes. In situ spectroscopic studies represent a particular essential upgrade, as they allow direct monitoring of dynamic electrochemical processes that ex situ methods cannot capture. However, the full potential of these techniques has yet to be thoroughly explored in the context of AMIBs. There is a significant opportunity to apply multimodal characterization techniques to analyze reaction intermediates and phase transitions. For AMIBs, present studies such as operando XAFS and Raman on sulfur anode and TOF–SIMS analysis of VO_2_ cathode have already provided valuable insights into phase transitions and co-insertion behavior. Techniques like in situ XRD are essential for monitoring the dynamic phase transitions during Mn deposition, while in situ Raman spectroscopy can offer real-time insights into the evolution of interfacial functional groups during charge and discharge cycles. Moreover, in situ diagnostics under realistic electrode thickness and electrolyte amount will be particularly valuable to capture pH gradients, by-product coverage, and interfacial instability that are otherwise underestimated in dilute, excess-electrolyte tests. Broader implementation of multimodal in situ methods in AMIBs will be essential to bridge electrochemical performance with real-time structural and chemical dynamics, ultimately guiding the rational design of durable and high-performance systems.
5. Integrating Artificial Intelligence (AI) and Machine Learning. AI-driven approaches have been validated to accelerate material and electrolyte screening when trained on standardized descriptors and comparable testing conditions. Machine learning models are expected effectively to uncover hidden structure–property relationships, guide rational electrode/electrolyte design, and mitigate Mn dissolution or dendrite formation. AI and machine learning are expected to drive a paradigm shift from “trial-and-error” to “intelligent design”, and provide an innovative pathway to address critical bottlenecks in Mn-ion storage field. However, the application of AI or ML to AMIBs is largely constrained by the scarcity of high-quality datasets and the lack of standardized experimental protocols at present. To address these challenges, one potential solution is generating synthetic datasets through advanced simulation techniques. The obtained simulation results will help complement experimental data and help train AI models. For instance, employing high-throughput phase-field simulations to generate extensive datasets, while utilizing machine learning models to establish predictive relationships between structural parameters and battery performance [113]. Meanwhile, the development of standardized experimental protocols, along with the integration of automated high-throughput experimentation, could also significantly accelerate the generation of large, high-quality datasets.

In summary, a realistic opportunity for AMIBs may lie in leveraging their potential for higher working voltages, as several reported studies have shown a higher operating voltage and capacity at high-voltage regions than Zn counterparts [32, 54, 62]. However, at the current stage, the decisive constraint for AMIBs is not the cathode-side capacity metrics but the anode-side reversibility in aqueous media. Bulk Mn metal is difficult to deploy directly in device formats, while Mn-powder anode has so far shown insufficient stability. Moreover, pursuing high-voltage operation in aqueous electrolyte inevitably increases the competition with hydrogen evolution, and for Mn anodes the severe water-splitting behavior can become an even more limiting factor than morphological instability. Therefore, near-term application niches should not assume immediate competitiveness with mature Zn-ion batteries in conventional cell formats. Instead, Mn-metal-free “rocking-chair” designs and supercapacitor-type configurations offer more pragmatic pathways before Mn metal reversibility and HER suppression are sufficiently addressed. This may enable acceptable energy output and safety for micro-power devices.

Research on AMIBs is relatively nascent with multiple bottlenecks yet considerable potential. Future progress will require not only isolated advances in electrodes, electrolytes, or interfaces, but the deliberate integration of these strategies into a coherent, system-level design. With increasing research investments, AMIBs will leverage their inherent advantages to emerge as a promising supplement for advanced energy storage applications [1, 114].
